# Grain protein content variation and its association analysis in barley

**DOI:** 10.1186/1471-2229-13-35

**Published:** 2013-03-03

**Authors:** Shengguan Cai, Gang Yu, Xianhong Chen, Yechang Huang, Xiaogang Jiang, Guoping Zhang, Xiaoli Jin

**Affiliations:** 1 Agronomy Department, Key Laboratory of Crop Germplasm Resource of Zhejiang Province, Zhejiang University, Hangzhou 310058, China; 2 Department of Nuclear Medicine, Second Affiliated Hospital of Zhejiang University School of Medicine, Hangzhou 310009, China; 3 Department of Life Science, Wenzhou Medical College, Wenzhou 325025, China

**Keywords:** Grain protein content, Malt quality, Molecular polymorphism, Tibetan wild barley (*Hordeum spontaneum* L.), Association mapping

## Abstract

**Background:**

Grain protein content (GPC) is an important quality determinant for barley used as malt, feed as well as food. It is controlled by a complex genetic system. GPC differs greatly among barley genotypes and is also variable across different environments. It is imperative to understand the genetic control of barley GPC and identify the genotypes with less variation under the different environments.

**Results:**

In this study, 59 cultivated and 99 Tibetan wild barley genotypes were used for a genome-wide association study (GWAS) and a multi-platform candidate gene-based association analysis, in order to identify the molecular markers associated with GPC. Tibetan wild barley had higher GPC than cultivated barley. The significant correlation between GPC and diastatic power (DP), and malt extract confirmed the importance of GPC in determining malt quality. Diversity arrays technology (DArT) markers associated with barley GPC were detected by GWAS. In addition, GWAS revealed two *HvNAM* genes as the candidate genes controlling GPC. No association was detected between *HvNAM1* polymorphism and GPC, while a single nucleotide polymorphism (SNP) (798, P < 0.01), located within the second intron of *HvNAM2*, was associated with GPC. There was a significant correlation between haplotypes of *HvNAM1*, *HvNAM2* and GPC in barley.

**Conclusions:**

The GWAS and candidate gene based-association study may be effectively used to determine the genetic variation of GPC in barley. The DArT markers and the polymorphism of *HvNAM* genes identified in this study are useful in developing high quality barley cultivars in the future. *HvNAM* genes could play a role in controlling barley GPC.

## Background

Grain protein content (GPC) is an important quality determinant in cereal crops. In barley, GPC is closely associated with feed and malt quality. Higher protein content is favorable for feed quality, while lower or moderate protein content is expected for malt barley. GPC affects malting quality in many ways, including yeast nutrition, haze formation in beer and enzyme activities [1,2].

Barley GPC is under polygenic control, with many quantitative trait loci (QTLs) having been mapped on all seven chromosomes, mainly on 2H, 4H, 5H and 6H [3,4]. All these loci had been determined by QTL mapping. Recently, genome-wide association study (GWAS) has been developed to dissect a variety of complex traits in plant [5,6]. GWAS has the advantage over the conventional QTL mapping in that GWAS can be performed on a number of genotypes. While a population used for conventional QTL mapping is developed from a bi-parental cross, only allowing the detection of a subset of loci/alleles within a plant and offering limited the resolution, due to insufficient recombination between the linked genetic loci. Hence, GWAS may present wider genetic variations and higher mapping resolution on phenotypes and traits at population level than conventional QTL mapping [6]. In barley, seven malt quality traits and some important agronomic traits have been effectively analyzed using GWAS [7-9].

Qinghai-Tibet Plateau, considered as one of the original centers of cultivated barley in the world, is rich in barley germplasm [10]. The polymorphism information content (PIC) value of Tibetan wild barley is higher than that of Chinese landraces according to analysis of SSR markers, and the wild barley has more unique alleles than the cultivated barley [11-13]. Thus, Tibetan wild barley is assumed to have wider variability in the genes controlling GPC [11-13]. Therefore, the population derived from Tibetan wild barley and cultivated barley worldwide could provide high resolution for GWAS in barley GPC.

A wheat QTL controlling GPC, named as *Gpc-B1*, was cloned, and a transcription factor (NAM-B1) was related to GPC by regulating senescence and protein remobilization [14]. Two orthologs genes (Genbank accession number DQ869678 and DQ869679) of *TtNAM-B1* in barley were identified on chromosomes 6H and 2H, respectively [14]. The single nucleotide polymorphism (SNP) analysis showed that allelic variation of the NAM-1 gene could be associated with GPC variation within the *Hordeum* genus. The differences in expression of *HvNAM-1* or other genes among barley cultivars or species could be attributed to GPC variation [15]. However, little research has been done regarding barley *HvNAM2* up to date,except that the sequence of *HvNAM2* was published [16].

The objectives of the current study are (1) to examine the correlation between GPC and malt quality; (2) to identify molecular markers associated with GPC in a barley mapping population by GWAS and determine the candidate genes controlling GPC; and (3) to analyze the association between *HvNAM* genes and GPC.

## Methods

### Plant materials

A collect of 158 barley accessions was used for association mapping and GPC analysis. These accessions included 59 barley cultivars (*H. vulgare* L.) from different areas of the world and 99 Tibetan wild barley (*H. spontaneum* L.). All barley cultivars and accessions were planted at the Huajiachi campus of Zhejiang University (Hangzhou, China, 120.0°E. 30.5°N) in the early winter of 2008 and 2009. Each accession was sown into a two-line plot, 2 m long and 0.24 m interval between the lines, and 40 seeds were planted in each line. All plots were supplied with 150 kg/ha of N, including 40 kg/ha of N as compound fertilizer applied before seeding, and 110 kg/ha of N as urea supplied at two-leaf stage and booting stage, respectively with equal amount. In addition, 180 kg/ha of potassium chloride was applied prior to seeding. The experiments were arranged in a block design with two replications. In each block, the 158 barley accessions were arranged randomly. All other agronomic managements, including weed and disease control, were the same as those applied locally. At seedling stage, leaves of each genotype were collected for DNA extract. The harvested seeds were stored at 4°C prior to malting. GPC and malt quality of all samples were measured, three measurements were done for each sample.

### GPC measurement

Mature grains were ground in a Cyclotec 1093 sample mill (Tecator AB, Hoganas, Sweden) and passed through a 0.5 mm screen. GPC was measured using the Kjeldahl method [17]. Protein content is calculated by duplicating a factor of 6.25 with N content.

### Malting and quality analysis

Grain samples (around 200 g) were micro-malted in a Micro-malting Apparatus (Phoenix System, Adelaide, Australia) using the following regime: 6 h steep, 14 h air-rest, 8 h steep, 14 h air-rest and 4 h steep, followed by 96 h germination – all performed at 15°C. The malts were then kilned at 65°C for 24 h, de-rooted and milled using a Tecator Cyclone mill fitted with a 0.5 mm screen. The soluble and total protein contents (SPC and TPC) in malt and the malt quality parameters (malt extract, Kolbach index, viscosity and DP) were determined according to the Analytica EBC Official Methods (European Brewery Convention, 1975).

### DNA extraction and genotypic analysis

Genomic DNA samples from young leaves of the barley seedlings were isolated as described by Uzunova et al. [18]. In brief, the leaf tissues were ground, and the resulting powder was re-suspended with CTAB (Hexadecyl trimethylammonium bromide) buffer (pH 5.0). To purify the DNA, insoluble particulates were removed through centrifugation. DNAs were precipitated from the aqueous phase and were washed thoroughly to remove contaminating salts.

Whole-genome profiling of DArT in all the DNA samples were analyzed using the Barley PstI (BstNI) version 1.7 array [19] at the Diversity Arrays Technology Pty Ltd in Australia. There are around 1,500 DArT markers, polymorphic in a wide range of barley cultivars, and 1,000 markers detected in wild barley accessions (http://www.triticarte.com.au/content/barley_diversity_analysis.html). Among the 1,576 reported markers and 1,319 polymorphic DArT markers, those with P value < 0.05 were used in the current study.

The primer pairs were designed using Primer3 [20] based on the *HvNAM1* and *HvNAM2* sequences (Genbank accession number DQ869678 and DQ869679, NCBI). 5^′^-atgggcagcccggactcatcctcc-3^′^ and 5^′^-tacagggattccagttcacgccggat-3^′^, 5^′^-atgggcagctcggactcatcttcc-3^′^ and 5^′^-tcagggattccagttcacgccgga-3^′^ were used for amplification of *HvNAM1* and *HvNAM2*, respectively. The PCR reaction mixture contains 20 mM Tris–HCl, 50 mM KCl, 2 mM MgCl_2_, l M of each dNTP, 5 pmol of each primer, 50–100 ng of genomic DNA and one unit of *Taq* DNA polymerase (Major-bio, Shanghai, China). The reaction was initially denatured at 95°C for 5 min, followed by 35 cycles of 95°C for 45 s, 60°C for 45 s and 72°C for 1.5 min. The PCR was terminated at 72°C for 10 min. The BigDye Terminator v3.1 cycle sequencing kit (Applied Biosystems, Foster City, CA, USA) was used for sequencing. The complete gene sequence was analyzed using Bioedit software (http://www.mbio.ncsu.edu/bioedit/bioedit.html).

### Data analysis

Pearson correlation analysis was conducted between GPC, SPC, TPC and malt quality parameters using SPSS 13.0 and SigmaPlot 10.0. Alignment of all the sequences was performed by ClustalW [21]. Genetic diversity was examined by 1319 randomly-distributed barley DArT markers over the genome at Diversity Arrays Technology Pty Ltd, Australia. The genetic polymorphism data from 1319 DArT markers were utilized to detect population structure by STRUCTURE software version 2.3.3 using an admixture model and five independent replicates of 100,000 Markov Chain iterations [22,23]. K values ranging from 1 to 10 were tested with a burn-in of 100,000 iterations and 100,000 Markov Chain Monte Carlo (MCMC) iterations according to the software’s instructions. The effect of population structure on GPC was tested using SAS GLM (SAS Institute, Cary, North Carolina, USA). The model included the components of the Q matrix obtained with STRUCTURE 2.2.3, which was used to illustrate population structure. R^2^ (variance explained by the model) was considered as an estimate of the proportion of phenotypic variation explained by population structure. The principal component analysis (PCA) was performed on the genotype data derived from 1319 DArT markers, which were standardized firstly using Unscrambler 9.7 (CAMO PROCESS AS, Oslo, Norway). *TASSEL* 2.01 was used to calculate linkage disequilibrium (LD) based on the parameter r^2^, which is a measurement of the correlation between a pair of variables [23]. The pair-wise relationship matrix (K-matrix), which was further employed for population correction in the association models, was calculated with 1319 DArT markers using *TASSEL 2.01*[23]. The two-year data of GPC were averaged for future association analysis. The structure-based association analysis with a K-matrix between DArT markers, *HvNAM* genes and GPC was calculated using *TASSEL 2.01*[23]. Association between DArT markers and the total trait variation was tested using mixed linear models (MLM), which was implemented in *TASSEL 2.01*. The P values were adjusted with permutation test using a step-down MinP procedure implemented in the *TASSEL 2.01*. The adjusted P value < 0.05 or <0.01 was considered as a criterion for association. The Manhattan plot of DArT markers and P value were drawn with the R software version 2.14.2 (http://www.r-project.org/). The association map was constructed using MapDraw version 2.1 [24].

Sequences of *HvNAM1* and *HvNAM2* were aligned using VectorNTI 10.0 (Invitrogen Corporation, Carlsbad, USA) or CLC main workbench 5 (CLC bio, Aarhus, Denmark), and alignments were edited manually using the BioEdit software. Haplotypes were inferred using the software *TASSEL 2.01*[23]. One barley accession was inferred as rare haplotypes and was excluded from further analysis. Grouped according to haplotypes in the *HvNAM* genes, GPC variation among the 59 cultivated and 99 Tibetan wild barley accessions was performed using the software SAS 9.0 software (SAS Institute, Cary, North Carolina, USA). For further association analysis between haplotype and GPC in the total 158 accessions, the SAS 9.0 software (SAS Institute, Cary, North Carolina, USA) was used to conduct analysis of variance (ANOVA) and multi-comparison analyses with least significant differences (LSD), the mean difference is significant at 0.05 level.

## Results

### The variation of protein content and Kolbach index

**Figure 1:**
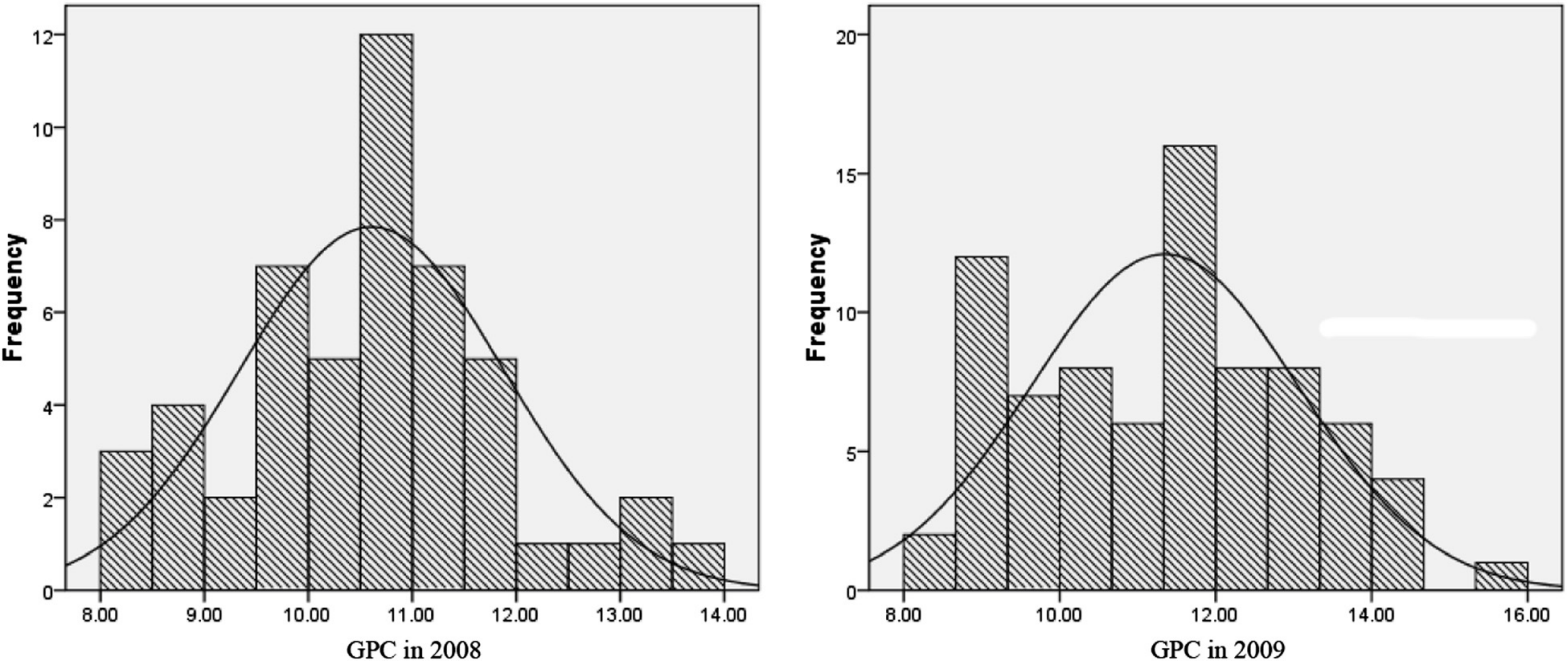
**Distribution of average grain protein content (GPC) in 2008 and 2009.** The X-axis shows the GPC in 2008 and 2009, the Y-axis shows the Number of individuals.

**Figure 2:**
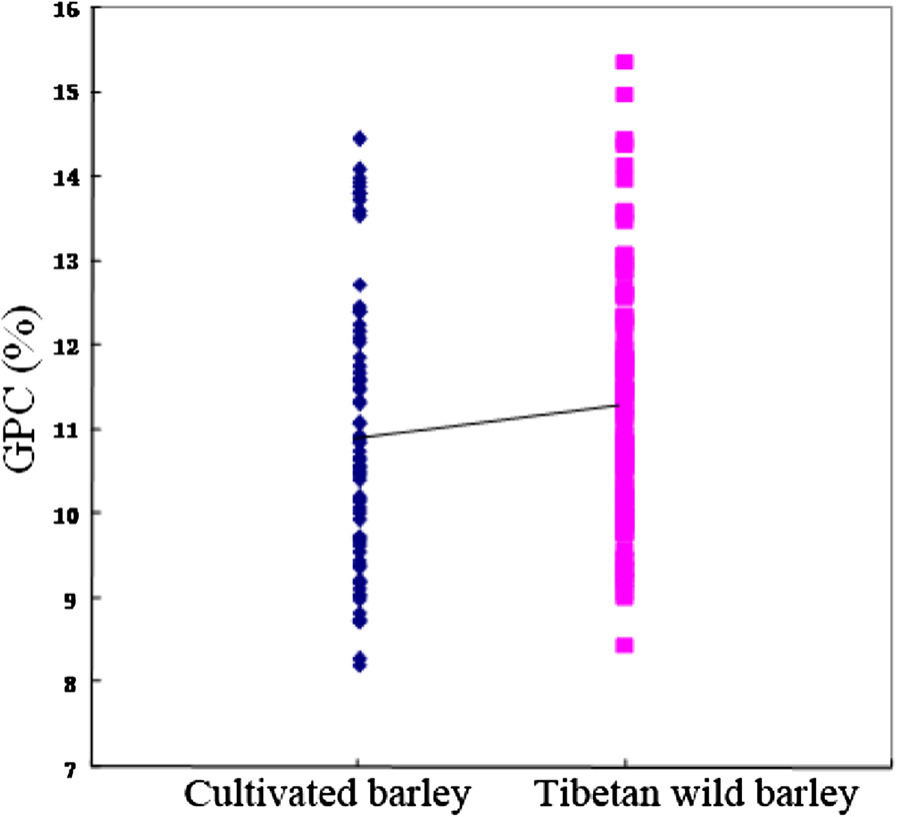
Grain protein content (GPC) in cultivated and Tibetan wild barleys.

**Figure 3:**
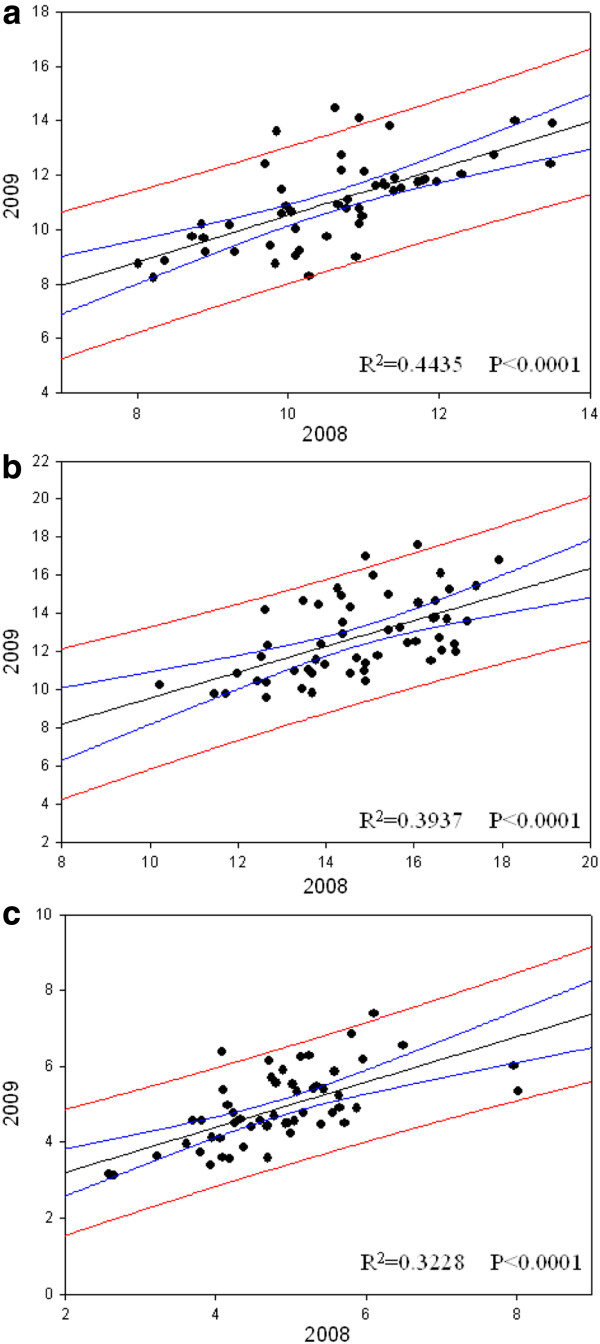
**Leverage plots of grain protein content (GPC) (a), total protein content in mat (TPC) (b) and soluble protein content in malt (SPC) (c) of cultivated barley in 2008 and 2009.** The X- and Y-axes show the data in 2008 and 2009, respectively.

The GPC in 59 cultivated and 99 Tibetan wild barley accessions ranged from 8.02% to 13.50% with a mean of 10.56% in 2008 and varied from 8.28% to 14.45% with a mean of 10.87% in 2009 (Figure 1). Overall, Tibetan wild barley had higher GPC than cultivated barley (Figure 2). Moreover, a normal distribution pattern of GPC is presented in Figure 1, suggesting multiple genes/QTLs control of GPC in barley. There was also a large variation in SPC, TPC and Kolbach index of the 158 accessions (Figure 3).

The values of GPC, SPC and TPC between 2008 and 2009 were significantly and positively correlated (R^2^ = 0.4435^**^ for GPC; R^2^ = 0.3937^**^ for SPC; R^2^ = 0.3937^**^ for TPC) (Figure 3), while the data of Kolbach index in 2008 could account for 55.11% of variation in 2009. Thus, it may suggest that GPC, SPC, TPC and Kolbach index are mainly controlled by genetic factors and also affected by environmental variation.

### The relationship between GPC, SPC, TPC and four malt quality parameters

The GPC presented the similar results in both years (Figure 3), so the mean values of two years were used in the correlation analysis. The results showed that GPC was significantly and positively correlated with SPC (0.628, P < 0.01), TPC (0.847, P < 0.01) and DP (0.340, P < 0.05), and negatively correlated with malt extract (−0.347, P < 0.01) (Table 1). There was no significant correlation between GPC and viscosity or Kolbach index. SPC was positively correlated with TPC (0.759, P < 0.01), Kolbach index (0.626, P < 0.01) and DP (0.456, P < 0.01), and negatively correlated with viscosity (−0.356, P < 0.01), indicating the significance of SPC in determining malt quality. Moreover, TPC was positively correlated with DP (0.465, P < 0.01) and negatively correlated with malt extract (−0.326, P < 0.01) (Table 1).

**Table 1:** The correlations between SPC (soluble protein content in malt), TPC (soluble protein content in malt), GPC (grain protein content), Kolbach index, DP (distatic power), malt extract and viscosity

	**GPC**	**SPC**	**TPC**
SPC	0.628**		
TPC	0.847**	0.759**	
Kolbach index	−0.031	0.626**	−0.003
DP	0.340*	0.456**	0.465**
Malt extract	−0.347**	−0.062	−0.326**
Viscosity	−0.055	−0.356**	−0.209

*P < 0.05, ** P < 0.01.

### Population structure and its impact on GPC variation

**Figure 4:**
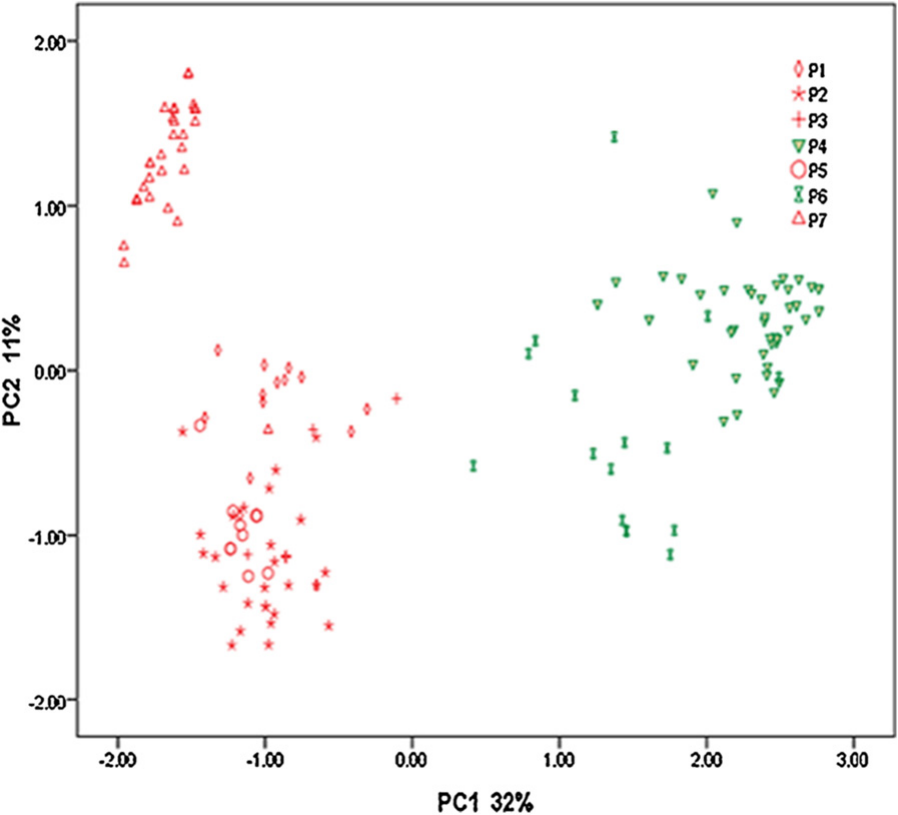
**Scatter plot of 59 cultivated and 99 Tibetan wild barley genotypes based on the first two principal component analysis (PCA) axes.** The percentage of variance explained by each axis is indicated. The red and green symbols represent Tibetan wild and cultivated barleys, respectively. P1 to P7 represent the subpopulation from 1 to 7, respectively.

One of the primary objectives in the current study was to determine the possibility whether GWAS could be used in association analysis of barley GPC and genetic markers. Hence, we obtained LD (*r*^2^) of the population used in this experiment. The extent of the obtained LD extended over 0.40 cM (Additional file 1: Figure S1), and 1319 DArT were distributed randomly over the whole barley genome, ensuring a good coverage of DArT markers on barley genome. The presence of population stratification and an unequal distribution of alleles within these groups could result in nonfunctional and spurious associations [25,26]. Thus, the population structure was taken into account in this study. The 1319 DArT markers were used to evaluate the subset of 59 cultivated and 99 Tibetan wild barley genotypes. Stratification within the barley population was detected by STRUCTURE and PCA. The highest likelihoods for sub-population (K values) calculated with STRUCTURE software were *K* = 7 (Additional file 2: Table S1, Additional file 3: Figure S2), indicating that seven sub-populations have the most stable variance. In addition, a PCA of the population structure was conducted. Interestingly, the cultivated and Tibetan wild barley were clearly separated into two groups with PCA (Figure 4), The cultivated barley accessions demonstrated a more distinct membership to subpopulation 4 and 6, while the Tibetan wild barley accessions belonged to subpopulation 1, 2, 3, 5 and 7 (Figure 4 and Additional file 4: Table S2). Collectively, these seven components accounted for 65.18% of the genetic variation. The first component accounted for 32% variation, while the second component explained 11% of the genetic variation (Figure 4). Then, a Q matrix with 7 sub-populations was used in the further analysis. Variance analysis of GPC data in 2008 and 2009 showed that population structure explains 10.6% of total variation, indicating the presence of impact of population structure on GPC.

### Association of DArT markers with GPC and determination of candidate genes

Generally, a stringent model may cause less spurious background association. In the current study, the structure-based association analysis with a K-matrix was calculated using *TASSEL 2.01*[23].

The two trials in 2008 and 2009 showed similar results; therefore, we combined the two year data and used the means for association analysis. The adjusted P values, obtained from step-down MinP procedure, were used for permutation test [27]. As the markers with adjusted P values < .05 are considered as significant, the probability of rejecting a single true null hypothesis across the entire set of hypotheses is held to <0.05. This test takes dependence between hypotheses into account and does not assume that hypotheses are independent as do other multiple test correction procedures [23]. Here, the association of DArT and GPC was shown in a Manhattan plot (Additional file 5: Figure S3). When the adjusted P value was <0.01, there were 3, 8, 1, 1 and 7 DArT markers, which were associated with GPC on 1H, 2H, 3H, 5H and 7H, respectively. Interestingly, five molecular markers in this study were close to the genetic markers of GPC reported previously (Table 2). Of them, bPb-1628 and bPb-1072 were close to marker HVBKASI, which was identified as *HvNAM2*[14]. In addition, bPb-8986 and bPb-3412 were close to the markers HVM36 and Bmag0751.

**Table 2:** The comparison between previously published and newly identified molecular markers in this study

**Published markers**	**Map**	**Proximal markers in this study**	**Position**	**Distance**	**Chromosome**	**QTL/gene**
ABG458	Hordeum-Consensus2006-DArT-6H	bPb-7179^*^	58.6	1.5	6H	*HvNAM1*(DQ869678)
ABG458	Hordeum-Consensus2006-DArT-6H	bPb-5822^*^	64.8	4.7	6H	*HvNAM1*(DQ869678)
ABG458	Hordeum-Consensus2006-DArT-6H	bPb-9522^*^	68.5	8.4	6H	*HvNAM1*(DQ869678)
MWG2029	Hordeum-Graner2-6H	HVM74^*^	66	4.5	6H	*HvNAM1*(DQ869678)
HVBKASI	Barley, B73xCPI-2H	bPb-2225^**^	67.6	4.1	2H	*HvNAM2*(DQ869679)
HVBKASI	Barley, B73xCPI-2H	bPb-1628^**^	67.6	4.1	2H	*HvNAM2*(DQ869679)
HVBKASI	Barley, B73xCPI-2H	bPb-1072^**^	67.6	4.1	2H	*HvNAM2*(DQ869679)
HVM36	Hordeum-Consensus2006-DArT-2H	bPb-8986^**^	26	4.2	2H	-
Bmag0751	Hv-Integrated2009-5H	bPb-3412^**^	45.6	0.3	5H	

*P < 0.05, ** P < 0.01.

**Figure 5:**
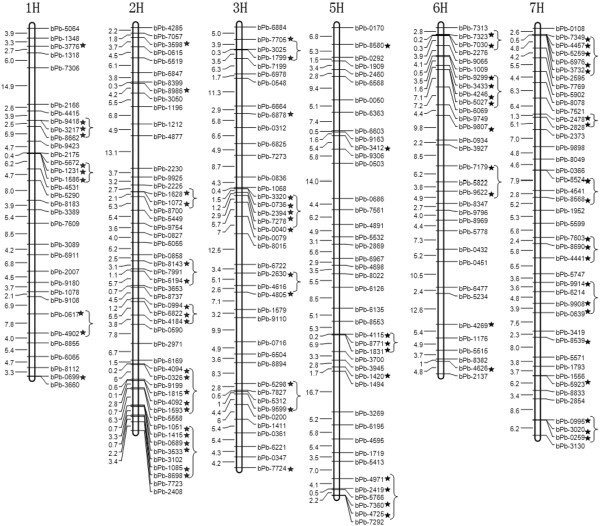
**The association map for grain protein content (GPC) in barley.** The map was constructed using MapDraw version 2.1 [20]. The asterisks denote the diversity arrays technology (DArT) markers associated with GPC. The brackets denote the DArT markers associated with GPC within 10 cM.

It was reported that the results of association analysis is affected by environmental factors [28,29]. Thus, a stringent criterion for significance, may bias studies against detection of causal associations that show significant Genotype-Environment interactions [30]. The correlation analysis of GPC between 2008 and 2009 showed that GPC were mainly controlled by genetic factors, but also affected by environmental conditions. Hence, we set the threshold of association analysis to 0.05, so as to detect possible markers associated with GPC. When the adjusted P value was <0.05, we found that GPC in barley was under polygenic control, and the relevant genes/QTLs were located on almost all chromosomes, except for 4H, mainly on chromosomes 2H and 7H (Figure 5). There were 10 DArT markers associated with GPC on chromosomes 1H and 5H, 20 on 2H, 13 on 3H, 11 on 6H, and 20 on 7H, respectively. The associated markers accounted for GPC variance ranged from 2.2 to 18.0%. Several DArT markers associated with GPC were closely localized within the genome. Thus, we considered the associated DArT markers within 10 cM to be the same locus. As a result, there were 5, 7, 6, 5, 6, and 8 loci on chromosome 1H, 2H, 3H, 5H, 6H, and 7H, respectively. Among those, a major QTL for GPC, which accounted for 40% of total variation, was quite close to the markers abg458, hvm74, and mwg2029, and it could be orthologous to the Gpc-B1 gene located on wheat chromosome 6BS. The *Gpc-B1* was associated with increased grain protein in wheat [1] and this QTL was identified as *HvNAM1* in barley [14] (Table 2). Similarly, in our study, the markers bPb-7179, bPb-5822 and bPb-9522 associated with GPC in barley were close to the markers abg458, hvm74, and mwg2029. In addition, the best Neighbor Joining tree showed that *HvNAM* genes have the closest distance with wheat NAM genes [14], and the colinearity of NAM locus between barley and wheat was also revealed [16]. Then, we inferred that *HvNAM* genes could be related to GPC in barley. Thus, *HvNAM1* and *HvNAM2* were chosen as the candidate genes for further association analysis of GPC.

### Association of *HvNAM* genes with GPC

**Figure 6:**
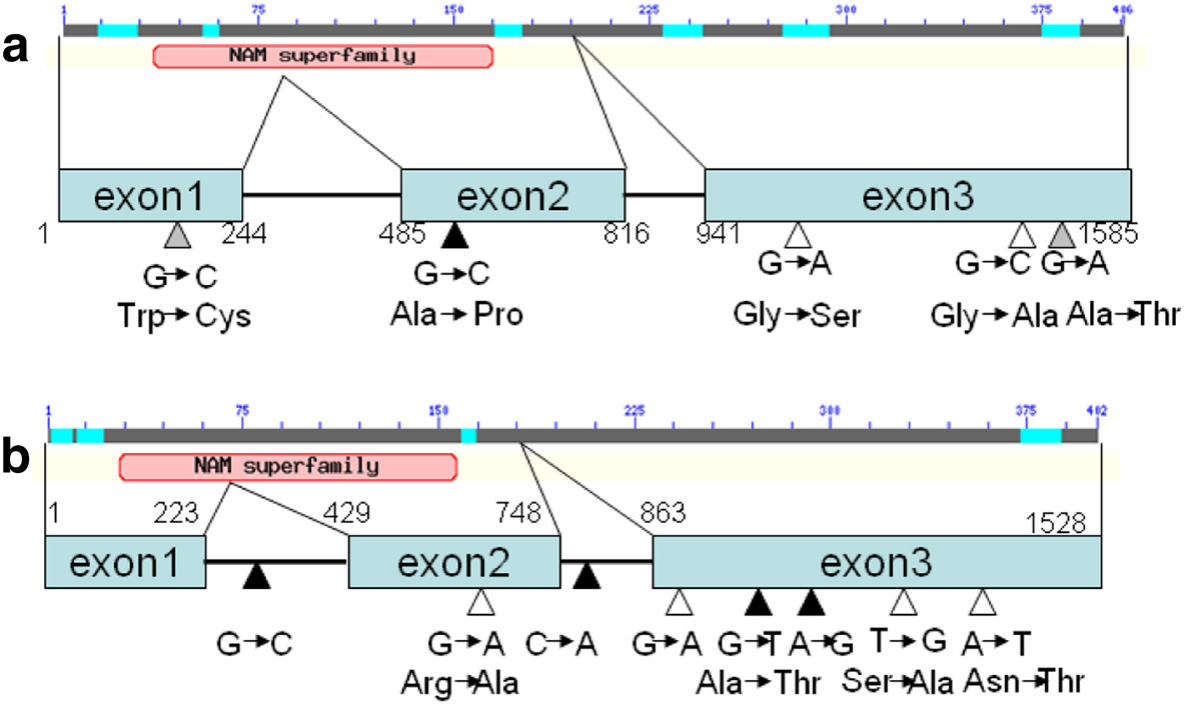
**Gene structure and single nucleotide polymorphisms (SNPs) of *HvNAM1 *and *HvNAM2*.** (**a**), Gene structure and SNPs of *HvNAM1*; (**b**), Gene structure and SNPs of *HvNAM2*. The boxes and lines represent exons and introns, respectively. The gray, white, and black triangles indicate the SNPs in cultivated, Tibetan wild and total barley genotypes, respectively.

**Figure 7:**
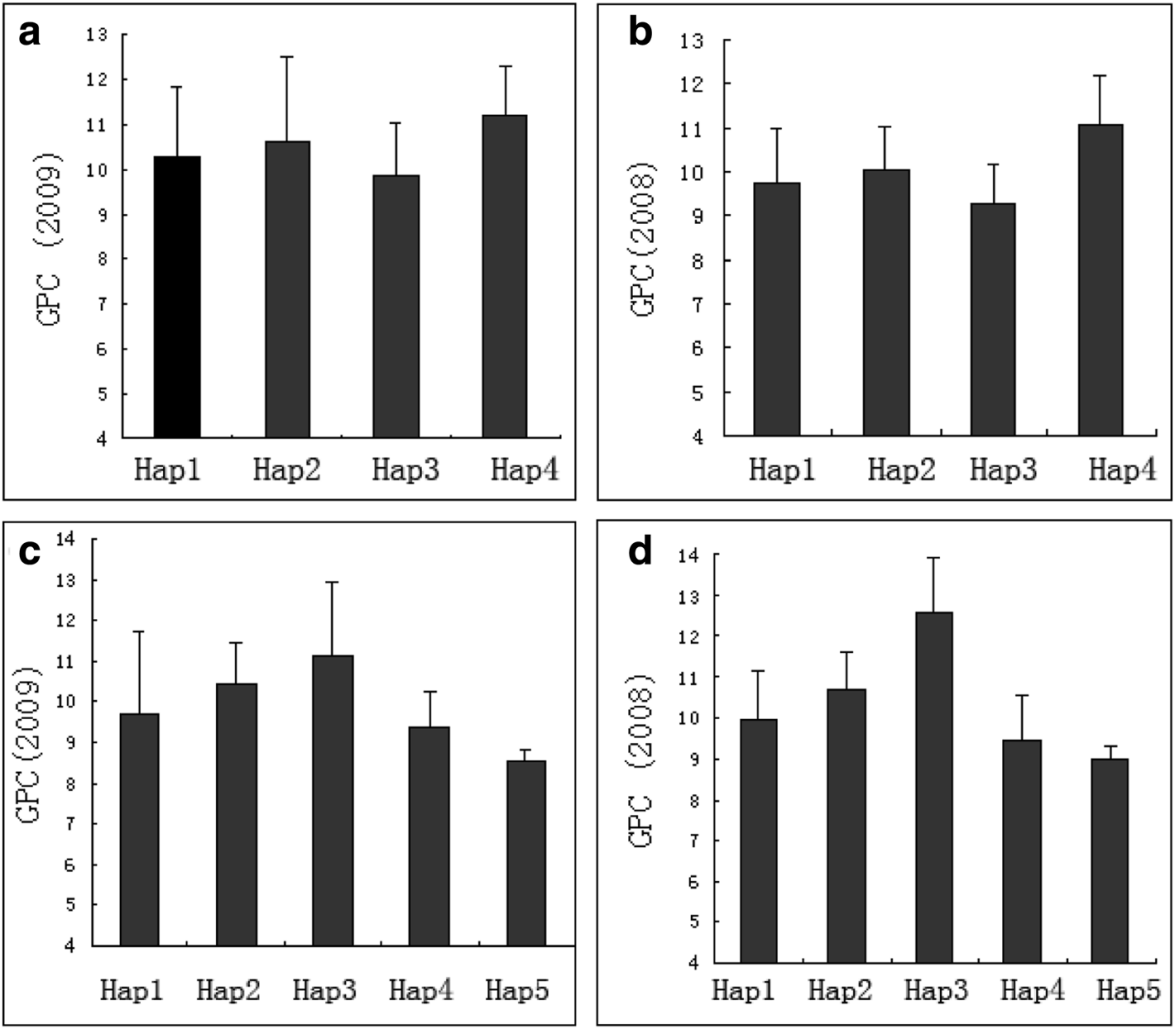
**Grain protein content (GPC) variation among 59 cultivated and 99 Tibetan wild barley accessions grouped according to the haplotypes in *HvNAM1 *(a, b) and *HvNAM2 *(c, d).** The means of GPC in 2009 (**a**, **c**) and 2008 (**b**, **d**) are shown. Error bars indicate standard deviations.

The sequences of the *HvNAM1* and *HvNAM2* genes were analyzed against the references from NCBI (accession number DQ869678 and DQ869679). The structure and SNPs of *HvNAM1* and *HvNAM2* are shown in Figure 6. The amplified length of *HvNAM1* gene was 1585 bp, containing 3 exons, 2 introns and a NAM super-family domain from amino acid 35 to 165. In comparison with the reference sequence (DQ869678), the *HvNAM1* in this study had five SNPs, located on bases 234, 544 and 1433 in cultivated barley, and on bases 544, 1190 and 1427 in Tibetan wild barley (Figure 6 and Additional file 6: Figure S4). All of the SNPs were within the coding region and resulted in 5 amino acid substitutions, where Trp, Ala, Gly, Gly, and Ala were replaced with Cys, Pro, Ser, Ala, and Thr, respectively. There was no association between *HvNAM1* polymorphism and GPC. Because no SNP of *HvNAM1* gene was found to be associated with GPC, haplotype-based association analysis was performed. Using the software *TASSEL 2.01* to infer haplotypes for *HvNAM1* gene among all accessions, we found five haplotypes within this gene. Three and 4 haplotypes were found in 59 cultivated and 99 Tibetan wild barley genotypes, respectively. There were one and two unique haplotypes in cultivated and Tibetan barley, respectively (Table 3). To analyze the possible differential effects of haplotypes on GPC, the population structure was taken into account. The haplotypes of *HvNAM1* explained 20.6% GPC variance in the tested population. As observed for the whole panel of accessions, the accessions carrying haplotype 4 of *HvNAM1* had the highest GPC, whereas accessions with haplotype 3 of *HvNAM1* had lowest GPC in two years (Figure 7).

**Table 3:** **The structure and nucleotide diversity of *HvNAM *** **genes in cultivated and Tibetan wild barleys**

**Gene**	**Cultivated barely**			**Tibetan wild barley**		
	**Total haplotypes**	**Shared haplotypes**	**Private haplotypes**	**Total haplotypes**	**Shared haplotypes**	**Private Haplotypes**
*HvNAM1* (DQ869678)	3	2	1	4	2	2
*HvNAM2* (DQ869679)	4	3	1	5	3	2

The amplified *HvNAM2* gene contained 3 exons and 2 introns with a NAM super-family domain between amino acids 28 and 157, and its length was 1528bp. The polypeptide sequence of *HvNAM2* showed 80% identity to that of *HvNAM1*. Eight SNPs were located on bases 307, 732, 798, 962, 979, 991, 1034 and 1289 in Tibetan wild barley, while 4 polymorphisms were present on bases 307, 798, 979 and 991 in the cultivated barley. Among these SNPs, SNP307 and SNP797 were within introns, while the others were within the coding sequence. SNP732, SNP979, SNP1034 and SNP1289 led to amino acid substitutions, specifically Arg, Ala, Ser and Asn replacement with Lys, Thr, Ala and Tyr, respectively. Six haplotypes could be classified according to polymorphisms among cultivated and Tibetan wild barley. Moreover, one haplotype in cultivated barley and 2 haplotypes in Tibetan wild barley were found unique (Table 3). The presence of new polymorphisms in Tibetan wild barley indicated that it could provide a new genetic resource in the genetic improvement of barley. However, only one SNP (798, P < 0.05) located within the second intron of *HvNAM2* (Figure 6 and Additional file 7: Figure S5) was associated with GPC as determined in two consecutive years in 59 cultivated and 99 Tibetan wild barley genotypes. Moreover, in order to analyze the effect of *HvNAM2* haplotypes on GPC in barley, one haplotype with one accession was excluded from the six haplotypes of *HvNAM2*. The haplotypes of *HvNAM2* explained 7.2% GPC variance in our population. We observed that the haplotype 3 of *HvNAM2* was higher in GPC, while the haplotype 5 of *HvNAM2* had the lowest GPC in both years (Figure 7).

## Discussion

Barley used for malting should have a GPC lower than 11.5%. GPC is influenced to a large extent by both genotype and environment [31,32]. In the current study, phenotyping of the diversity panel provided some valuable information about the range and distribution of GPC in barley. Genotype and environment interactions are indeed apparent when comparing the GPC data over the two consecutive years. Our results showed that some Tibetan wild accessions with higher GPC could be useful for breeding both feed and food barley cultivars. Although there were significant differences in GPC, SPC and TPC among genotypes over the two consecutive years, the traits were mainly controlled by genetic factors as indicated by their high consistency over the two years.

A negative correlation between GPC and malt extract and a positive correlation between GPC and DP have been reported [33]. Similarly, in the current study, we found that TPC was negatively correlated with malt extract and positively correlated with DP. Interestingly, SPC was correlated with all malt quality parameters except malt extract. Obviously, the protein content in both grain and malt is closely related to malt quality. Therefore, it is imperative for us to develop barley varieties with stable GPC in malt barley breeding.

The advantages of GWAS over the conventional QTL mapping, based on a population from a bi-parental cross have been confirmed [34]. Compared to QTL mapping, GWAS increases the range of natural variation that can be surveyed in a single experiment and the number of significant regions that are likely to be identified [12]. Hence, GWAS could provide higher resolution than QTL mapping, and facilitate fine-mapping and gene discovery. The materials used in our GWAS study, included 59 worldwide cultivated and 99 Tibetan wild barley accessions, which cover representative accessions from most of the barley-growing regions in the world.

GPC were mainly controlled by genetic factors and also affected by environmental variation according the correlation analysis. However, a stringent criterion for significance, may bias studies against detection of causal associations that show significant Genotype-Environment interactions [30]. Thus, we chose 0.01 and 0.05 as the threshold of association analysis, in order to detect possible markers associated with GPC. As a result, GWAS identified as many as 5, 7, 6, 5, 6 and 8 loci to be associated with barley GPC on chromosomes 1H, 2H, 3H, 5H, 6H and 7H, respectively. These results showed that many more molecular markers associated with GPC could be detected by GWAS than by conventional QTL mapping.

In addition to the discovery of the DArT markers for GPC, the completion of the association map for GPC is a significant step towards the cloning of GPC related genes. The identified markers for GPC will be very useful in the evaluation and screening of barley accessions with reasonable GPC. In comparison with previous studies [1,4,15,31], we found more markers in this study, including 3, 3, and 1 marker(s) on chromosome 6H, 2H and 5H, respectively (Table 2). Three major QTLs were identified on chromosomes 6H and 2H using a barley mapping population developed from a cross between ‘Karl’, a low grain protein six-rowed variety and ‘Lewis’, a high grain protein two-rowed variety. The three QTLs could explain 56% of the total heritable variance of GPC [1]. Two of them were identified as the *HvNAM1* and *HvNAM2* genes in barley, the homologs of a NAC transcription factor (NAM-B1) that increases GPC by regulating senescence in wheat [14]. Therefore, we considered *HvNAM1* and *HvNAM2* as the candidate genes controlling GPC. Due to the effect of gene-target association to identify SNP markers for use in barley [35], the association between two candidate genes, *HvNAM1* and *HvNAM2*, and GPC was analyzed, in order to examine the genetic architecture of GPC and to identify GPC loci in barley.

Jamar et al. found that allelic variation of the functional NAM-1 gene could be associated with GPC variation within the genus *Hordeum*[15], and the 13 genotypes used in their study could be classified into three haplotypes: 11 European varieties of *H. vulgare* being gathered as haplotype 1, one *H. spontaneum* (Hs) and one *Hordeum bulbosum* (Hb) being classified as haplotype 2 (Genbank accession number EU908210) and haplotype 3 (Genbank accession number EU908211), respectively. By comparing to the reference sequence (DQ869678), 3 SNPs were identified on bases 355, 483 and 554 of *HvNAM1*. However, we did not identify these SNPs in the current study. Instead, we found 3 SNPs located on bases 234, 544 and 1433 in the cultivated barley and 3 SNPs on bases 544, 1190 and 1427 in Tibetan wild barley. No association was detected between the polymorphisms of *HvNAM1* and GPC, however there was significant correlation between *HvNAM1* haplotypes and GPC. Moreover, eight SNPs within *HvNAM2* were located on bases 307, 732, 798, 962, 979, 991, 1034 and 1289 in the Tibetan wild barley, but only 4 SNPs were present on bases 307, 798, 979 and 991 in the cultivated barley. Interestingly, a single SNP (798, P < 0.05) within *HvNAM2* gene, located on the second intron, was associated with GPC. To gain further insight, the correlation between *HvNAM2* haplotypes and GPC was analyzed in barley, where The DArT markers close to *HvNAM1 and HvNAM2* explained 18% and 6.4% GPC variance, while the haplotypes of *HvNAM1 and HvNAM2* accounted for 20.6% and 7.2% of GPC variance, respectively. The comprehensive analysis, including the primary GWAS, the colinearity of NAM locus between barley and wheat, the best Neighbor Joining tree of NAM genes in Arabidopsis and other crops and the association analysis of *HvNAM* genes, indicated that *HvNAM* genes could drive the variation in barley GPC. Moreover, the results also showed that the adjusted P value < 0.05 could be reasonable for finding the molecular markers associated with traits which are greatly affected by environmental factors. In fact, the threshold with P <0.05 used in our primary GWAS of GPC ensured identification of the DArT markers, which were not detected in the analysis with the threshold of P <0.01. One of candidate genes, *HvNAM1*, detected in the association analysis with adjusted P values <0.05, was found to be associated with GPC. The current results indicate the suitability of the adjusted P value <0.05 for identifying the molecular markers associated with GPC. Similarly, the adjusted P values <0.05 was used as the criteria for association analysis in other research [36].

Ultimately, the identification of SNPs and haplotypes of *HvNAM* genes could enable the development of useful molecular markers for GPC. Here, the association analysis may provide some molecular markers of *HvNAM* genes with potential importance for the early selection in malt barley breeding.

More importantly, it will shed some light on the molecular mechanisms responsible for the genotypic differences of GPC in cultivated and wild barley. Furthermore, the exact chromosome regions of these markers would be interesting for researchers to understand the genetics of GPC, since most of these regions have been not annotated in terms of their function. However, association mapping only provides statistical and indirect evidences for the function of identified genes, so we are targeting some direct evidences into the underlying molecular mechanisms of GPC and malting quality in future research.

## Conclusions

This study has demonstrated close correlation between protein content and malt quality parameters, indicating that it is imperative for us to develop barley varieties with a stable GPC. The identified markers for GPC in this study will be very useful in evaluation and screening of barley germplasm with reasonable GPC. Moreover, the haplotypes of *HvNAM1* and *HvNAM2*, SNP and DArT markers, which were associated with GPC in barley, could provide key molecular markers for the selection of malt quality traits. In addition, GWAS is very useful for finding candidate genes and may provide a powerful tool for identifying the different loci influencing GPC in barley.

## Competing interests

The authors declare that they have no competing interest.

## Authors’ contributions

Conceived and designed the experiments: XJ. Performed the experiments: XJ, SC, YH, XC, XJ. Analyzed the data: XJ. Contributed reagents/materials/analysis tools: XJ, GZ. Wrote the paper: XJ, GZ. All authors read and approved the final manuscript.

## Supplementary Material

Additional file 1: Figure S1 Decay of linkage disequilibrium of the population of 158 accessions based on 1319 DArT markers. The equation of LD decay was y = −0.01ln(x) + 0.091, the decay of genetic distance is 0.40 cM (r^2^ = 0.1). The X-axis showed that the genetic distance, The Y-axis showed the r^2^, the squared allele frequency correlations, which is a measurement of the correlation between a pair of variables. Click here for file

Additional file 2: Table S1 Summary of the logarithm of probability of data likelihoods (*LnP(D)*) for population structure of genome-wide association study (GWAS) in assessed barley genotypes. Note: Ln p(D), Natural logarithm of the probability of data. Likelihoods were calculated over ten independent runs of a burn-in of 100,000 iterations, followed by 100,000 iterations of using a model allowing for no admixture and correlated allele frequencies. *K* value was set up from 1 to 10 and 1319 DArT markers were used in this analysis. Click here for file

Additional file 3: Figure S2 Population structure of 59 cultivated and 99 Tibetan wild barley accessions based on the genetic diversity detected by 1319 DArT markers. P1 to P7 represent the seven subpopulations. Click here for file

Additional file 4: Table S2 Population sub-structuring in the 158 barley accessions. Note: C and W represent the cultivated barley and Tibetan wild barley, respectively. Click here for file

Additional file 5: Figure S3. The Manhattan plot of DArT markers used in association analysis. The DArT markers with unknown genetic location were excluded from the Manhattan plot. The P values were adjusted with permutation test using a step-down MinP procedure. 1H to 7H on the X-axis denoted the barley chromosomes from 1H to 7H, respectively. The Y-axis showed that the –Log_10_(P), The two dashed lines indicate the P value = 0.05 and 0.01. Click here for file

Additional file 6: Figure S4 Multiple sequence alignment of *HvNAM-1* gene for different haplotypes. The symbols under the sequence alignment indicate identical residues (*), and strongly conserved (:) and weakly conserved (.) substitutions by CLUSTALW (http://align.genome.jp/). Nucleotides belong to exon are shaded in gray. The SNPs are marked in red. Click here for file

Additional file 7: Figure S5 Multiple sequence alignment of *HvNAM-2* gene for different haplotypes. The symbols under the sequence alignment indicate identical residues (*), and strongly conserved (:) and weakly conserved (.) substitutions by CLUSTALW (http://align.genome.jp/). Nucleotides belong to exon are shaded in gray. The SNPs are marked in red. Click here for file

## References

[B1] See DK Kephart V Blake K Mapping genes controlling variation in barley grain protein concentration Crop Sci 2002 42 680 685 10.2135/cropsci2002.0680

[B2] Clancy JA Han F Ullrich SE Comparative mapping of-amylase activity QTLs among three barley crosses. North American barley genome project Crop Sci 2003 43 1043 1052 10.2135/cropsci2003.1043

[B3] Ullrich SE Slafer GA, Molina-Cano JL, Savin R, Araus JL, Romagosa I Genetics and breeding of barley feed quality attributes Barley Science: Recent Advances from Molecular Biology to Agronomy of Yield and Quality 2002 Food Products Press: Food Products Press 115 142

[B4] Emebiria LC Moodya DB Horsleyb R Panozzoa J Read BJ The genetic control of grain protein content variation in a doubled haploid population derived from a cross between Australian and North American two-rowed barley lines J Cereal Sci 2005 41 107 114 10.1016/j.jcs.2004.08.012

[B5] Polanda JA Bradbury PJ Buckler ES Nelson RJ Genome-wide nested association mapping of quantitative resistance to northern leaf blight in maize Proc Natl Acad Sci USA 2010 108 6893 6899 10.1073/pnas.1010894108 PMC3084105 21482771

[B6] Hu VW Addington A Hyman A Novel autism subtype-dependent genetic variants are revealed by quantitative trait and subphenotype association analyses of published GWAS data PLoS One 2011 6 4 e19067 10.1371/journal.pone.0019067 21556359 PMC3083416

[B7] Beattie AD Edney MJ Scoles GJ Rossnagel BG Association Mapping of Malting Quality Data from Western Canadian Two-row Barley Cooperative Trials Crop Sci 2010 50 1649 1663 10.2135/cropsci2009.06.0334

[B8] Pasam RK Sharma R Malosetti M Van Eeuwijk FA Haseneyer G Kilian B Graner A Genome-wide association studies for agronomical traits in a world wide spring barley collection BMC Plant Biol 2012 12 16 10.1186/1471-2229-12-16 22284310 PMC3349577

[B9] Stracke S Haseneyer G Veyrieras JB Geiger HH Sauer S Graner A Piepho HP Association mapping reveals gene action and interactions in the determination of flowering time in barley Theor Appl Genet 2009 118 259 273 10.1007/s00122-008-0896-y 18830577

[B10] Glémin S Bataillon TA Comparative view of the evolution of grasses under domestication New Phytol 2009 183 273 290 10.1111/j.1469-8137.2009.02884.x 19515223

[B11] Nevo E Shewry PR Origin, evolution, population genetics and resources for breeding of wild barley, *Hordeum spontaneum*, in the Fertile Crescent Barley: Genetics 1992 Molecular Biology and Biotechnology, CAB International, Wallingford: Biochemistry 19 43

[B12] Jin XL Cai SG Han Y Wang J Wei K Zhang GP Genetic variants of *HvGlb1* in Tibetan annual wild barley and cultivated barley and their correlation with malt quality J Cereal Sci 2011 53 59 64 10.1016/j.jcs.2010.09.006

[B13] Qiu L Wu DZ Ali S Cai SG Dai F Jin XL Wu FB Zhang GP Evaluation of salinity tolerance and analysis of allelic function of *HvHKT1* and *HvHKT2* in Tibetan wild barley Theor Appl Genet 2011 122 695 703 10.1007/s00122-010-1479-2 20981400

[B14] Uauy C Distelfeld A Fahima T Blechl A Ubcovsky J A NAC gene regulating senescence improves grain protein, zinc, and iron content in wheat Science 2006 314 1298 1301 10.1126/science.1133649 17124321 PMC4737439

[B15] Jamar C Loffet F Frettinger P Ramsay L Fauconnier M-L Jardin P *NAM-1* gene polymorphism and grain protein content in Hordeum J Plant Physiol 2010 167 497 501 10.1016/j.jplph.2009.10.014 20005003

[B16] Distelfeld A Korol A Dubcovsky J Uauy C Blake T Fahima T Colinearity between the barley grain protein content (GPC) QTL on chromosome arm 6HS and the wheat Gpc-B1 region Mol Breeding 2008 22 25 38 10.1007/s11032-007-9153-3

[B17] Kjeldahl JZ A new method for the determination of nitrogen in organic matter Anal Chem 1983 22 366 382

[B18] Uzunova M Ecke W WeiBleder K Röbbelen G Mapping the genome of rapeseed (*Brassica napus* L.). I. Construction of an RFLP linkage map and localization of QTLs for seed glucosinolate content Theor Appl Genet 1995 90 194 204 10.1007/BF00222202 24173891

[B19] Wenzl P Carling K Kudrna D Jaccoud D Huttner E Kleinhofs A Kilian A Diversity Arrays Technology (DArT) for whole-genome profiling of barley Proc Natl Acad Sci USA 2004 101 9915 9920 10.1073/pnas.0401076101 15192146 PMC470773

[B20] Rozen S, Skaletsky HJ, Krawetz S, Misener S Primer3 on the WWW for general users and for biologist programmers. Bioinformatics methods and protocols: methods in molecular biology 2000 Totowa: Humana Press 365 386 10.1385/1-59259-192-2:365 10547847

[B21] Thompson JD Higgins DG Gibson TJ CLUSTAL W: improving the sensitivity of progressive multiple sequence alignment through sequence weighting, position-specific gap penalties and weight matrix choice Nucl Acid Res 1994 22 4673 4680 10.1093/nar/22.22.4673 PMC308517 7984417

[B22] Hubisz MJ Falush D Stephens M Pritchard JK Inferring weak population structure with the assistance of sample group information Mol Ecol Resour 2009 9 1322 1332 10.1111/j.1755-0998.2009.02591.x 21564903 PMC3518025

[B23] Pritchard JK Stephens M Donnelly P Inference of population structure using multilocus genotype data Genetics 2000 155 945 959 10835412 10.1093/genetics/155.2.945 PMC1461096

[B24] Liu RH Meng JL MapDraw: a Microsoft Excel macro for drawing genetic linkage maps based on given genetic linkage data Heraditas (Beijing) 2003 25 317 321 15639879

[B25] Knowler WC Williams RC Pettitt DJ Steinberg AG Gm3–5, 13, 14 and type 2 diabetes mellitus: an association in American Indians with genetic admixture Am J Hum Genet 1988 43 520 526 3177389 PMC1715499

[B26] Sharbel TF Haubold B Mitchell-Olds T Genetic isolation by distance in Arabidopsis thaliana: biogeography and postglacial colonization of Europe Mol Ecol 2000 9 2109 2118 10.1046/j.1365-294X.2000.01122.x 11123622

[B27] Ge YC Dudoit S Speed TP Resampling-based Multiple Testing for Microarray. Data Analysis Sociedad de Estadistica e Investigaci6n Operativa Test 2003 12 1 1 77

[B28] Chan EK Rowe HC Hansen BG Kliebenstein DJ The complex genetic architecture of the metabolome PLoS Genet 2010 6 e1001198 10.1371/journal.pgen.1001198 21079692 PMC2973833

[B29] Manolio TA Collins FS Cox NJ Goldstein DB Hindorff LA Finding the missing heritability of complex diseases Nature 2009 461 747 753 10.1038/nature08494 19812666 PMC2831613

[B30] Liu YJ Papasian CJ Liu JF Hamilton J Deng HW Is replication the gold standard for validating genome-wide association findings? PLoS One 2008 3 e4037 10.1371/journal.pone.0004037 19112512 PMC2605260

[B31] Jukanti AK Fischer AM A high-grain protein content locus on barley (*Hordeum vulgare*) chromosome 6 is associated with increased flag leaf proteolysis and nitrogen remobilization Physiol Plant 2008 132 426 439 10.1111/j.1399-3054.2007.01044.x 18333996

[B32] Smith DB Barley seed protein and its effects on malting and brewing quality Plant Variety Seed 1990 3 63 80

[B33] Wang JM Zhang GP Chen JX Ding SR Zhou TY Variation of grain and malt qualities in barley as affected by cultivars and environments Agr Sci in China 2003 2 699 705

[B34] Jin XL Wei K Zhang GP A genome-wide association analysis of quantitative trait loci for protein fraction content in Tibetan wild barley Biotechnol Lett 2012 34 159 165 10.1007/s10529-011-0736-z 21915716

[B35] Yan JB Kandianis CB Harjes CE Bai L Kim EH Yang XH Skinner DJ Fu ZY Mitchell S Li Q Fernandez MGS Zaharieva M Babu R Fu Y Palacios N Li JS Penna D Brutnell T Buckler ES Warburton ML Rocheford T Rare genetic variation at *Zea mays* crtRB1 increases β-carotene in maize grain Nat Genet 2010 42 322 327 10.1038/ng.551 20305664

[B36] Wang N Qian W Suppanz D Wei LJ Mao BZ Long Y Meng JL Müller AE Jung C Flowering time variation in oilseed rape (*Brassica napus* L.) is associated with allelic variation in the FRIGIDA homologue *BnaA. FRI.a* J Exp Bot 2011 62 15 5641 5658 10.1093/jxb/err249 21862478 PMC3223056

