# Supplementary figures and images for: Grain protein content variation and its association analysis in barley
Source: BMC Plant Biol. 2013 Mar 3;13:35. doi: 10.1186/1471-2229-13-35 (PMC3608362; doi:10.1186/1471-2229-13-35)

**Fig. S1**


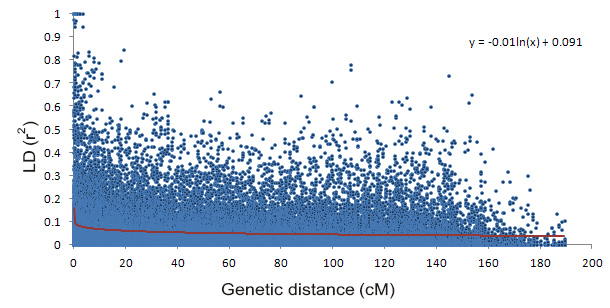

Supplement: Additional file 1: Figure S1 — Decay of linkage disequilibrium of the population of 158 accessions based on 1319 DArT markers. The equation of LD decay was y = −0.01ln(x) + 0.091, the decay of genetic distance is 0.40 cM (r2 = 0.1). The X-axis showed that the genetic distance, The Y-axis showed the r2, the squared allele frequency correlations, which is a measurement of the correlation between a pair of variables. [file 1471-2229-13-35-S1.doc]

## Fig. S2


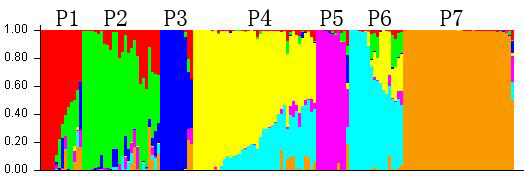

Supplement: Additional file 3: Figure S2 — Population structure of 59 cultivated and 99 Tibetan wild barley accessions based on the genetic diversity detected by 1319 DArT markers. P1 to P7 represent the seven subpopulations. [file 1471-2229-13-35-S3.doc]

**Fig S3**

**
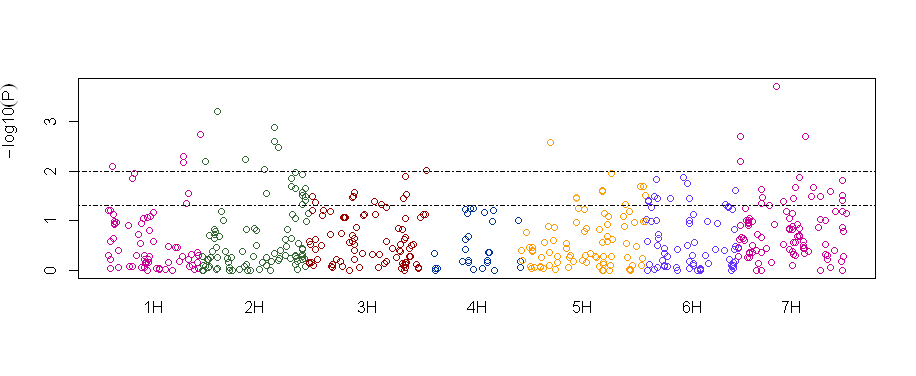
**

Supplement: Additional file 5: Figure S3. — The Manhattan plot of DArT markers used in association analysis. The DArT markers with unknown genetic location were excluded from the Manhattan plot. The P values were adjusted with permutation test using a step-down MinP procedure. 1H to 7H on the X-axis denoted the barley chromosomes from 1H to 7H, respectively. The Y-axis showed that the –Log10(P), The two dashed lines indicate the P value = 0.05 and 0.01. [file 1471-2229-13-35-S5.doc]
