# Supplementary material for: Grain protein content variation and its association analysis in barley
Source: BMC Plant Biol. 2013 Mar 3;13:35. doi: 10.1186/1471-2229-13-35 (PMC3608362; doi:10.1186/1471-2229-13-35)
Supplement: Additional file 2: Table S1 — Summary of the logarithm of probability of data likelihoods (LnP(D)) for population structure of genome-wide association study (GWAS) in assessed barley genotypes. Note: Ln p(D), Natural logarithm of the probability of data. Likelihoods were calculated over ten independent runs of a burn-in of 100,000 iterations, followed by 100,000 iterations of using a model allowing for no admixture and correlated allele frequencies. K value was set up from 1 to 10 and 1319 DArT markers were used in this analysis. [file 1471-2229-13-35-S2.doc]

**Table S1**

Summary of the logarithm of probability of data likelihoods (*LnP(D)*) for population structure of genome-wide association study (GWAS) in assessed barley genotypes.

| ***K*** | **Ln p(D)** | **SD** |
| --- | --- | --- |
| 1 | -115804.1 | 635.8 |
| 2 | -95429.7 | 1291.1 |
| 3 | -84947.6 | 1889.4 |
| 4 | -78275.6 | 2502.2 |
| 5 | -74194.9 | 3176.8 |
| 6 | -70483.4 | 4495.6 |
| 7 | -68498.1 | 28525.5 |
| 8 | -75595.5 | 48906.6 |
| 9 | -112957.2 | 5130.5 |
| 10 | -82425.2 | 6945.7 |

Note: Ln p(D), Natural logarithm of the probability of data. Likelihoods were calculated over ten independent runs of a burn-in of 100,000 iterations, followed by 100,000 iterations of using a model allowing for no admixture and correlated allele frequencies. *K* value was set up from 1 to 10 and 1319 DArT markers were used in this analysis.
