# Supplementary material for: Grain protein content variation and its association analysis in barley
Source: BMC Plant Biol. 2013 Mar 3;13:35. doi: 10.1186/1471-2229-13-35 (PMC3608362; doi:10.1186/1471-2229-13-35)
Supplement: Additional file 4: Table S2 — Population sub-structuring in the 158 barley accessions. Note: C and W represent the cultivated barley and Tibetan wild barley, respectively. [file 1471-2229-13-35-S4.doc]

**Table S2**

Population sub-structuring in the 158 barley accessions.

| subpopulation  No. | Accession | C/W | *HvNAM1*  Haplotype | *HvNAM2*  Haplotype | subpopulation  No. | Accession | C/W | *HvNAM1*  Haplotype | *HvNAM2*  Haplotype |
| --- | --- | --- | --- | --- | --- | --- | --- | --- | --- |
| 1 | T003 | W | Hap1 | Hap1 | 4 | Fuyin1hao | C | Hap1 | Hap1 |
| 1 | T010 | W | Hap1 | Hap1 | 4 | Haha | C | Hap3 | Hap2 |
| 1 | T011 | W | Hap1 | Hap1 | 4 | Donggongdamai | C | Hap1 | Hap1 |
| 1 | T012 | W | Hap1 | Hap2 | 4 | Belae | C | Hap2 | Hap1 |
| 1 | T014 | W | Hap2 | Hap2 | 4 | Allaweinuw | C | Hap2 | Hap1 |
| 1 | T015 | W | Hap1 | Hap1 | 4 | Rudong3hao | C | Hap1 | Hap1 |
| 1 | T016 | W | Hap1 | Hap3 | 4 | Qingyin2916 | C | Hap1 | Hap1 |
| 1 | T027 | W | Hap2 | Hap1 | 4 | Qingyin3506 | C | Hap1 | Hap2 |
| 1 | T030 | W | Hap2 | Hap3 | 4 | Mianyang81-18 | C | Hap1 | Hap1 |
| 1 | T033 | W | Hap1 | Hap1 | 4 | Hongguangerleng | C | Hap3 | Hap2 |
| 1 | T034 | W | Hap1 | Hap1 | 4 | Zhongshu1hao | C | Hap1 | Hap2 |
| 1 | T066 | W | Hap1 | Hap1 | 4 | Daimai1hao | C | Hap1 | Hap1 |
| 1 | T070 | W | Hap1 | Hap1 | 4 | Ganpi1hao | C | Hap3 | Hap2 |
| 1 | T084 | W | Hap1 | Hap1 | 4 | Zhenongda3hao | C | Hap1 | Hap2 |
| 2 | T001 | W | Hap1 | Hap1 | 5 | T018 | W | Hap1 | Hap1 |
| 2 | T002 | W | Hap1 | Hap1 | 5 | T038 | W | Hap1 | Hap2 |
| 2 | T004 | W | Hap4 | Hap1 | 5 | T043 | W | Hap2 | Hap2 |
| 2 | T006 | W | Hap1 | Hap2 | 5 | T049 | W | Hap2 | Hap1 |
| 2 | T007 | W | Hap1 | Hap1 | 5 | T050 | W | Hap2 | Hap1 |
| 2 | T008 | W | Hap2 | Hap1 | 5 | T052 | W | Hap1 | Hap1 |
| 2 | T009 | W | Hap4 | Hap6 | 5 | T055 | W | Hap1 | Hap1 |
| 2 | T013 | W | Hap2 | Hap1 | 5 | T063 | W | Hap1 | Hap1 |
| 2 | T017 | W | Hap2 | Hap2 | 5 | T083 | W | Hap1 | Hap1 |
| 2 | T019 | W | Hap2 | Hap2 | 5 | T098 | W | Hap1 | Hap1 |
| 2 | T020 | W | Hap2 | Hap1 | 5 | T103 | W | Hap4 | Hap3 |
| 2 | T021 | W | Hap2 | Hap1 | 6 | Ludaomai | C | Hap2 | Hap4 |
| 2 | T022 | W | Hap2 | Hap1 | 6 | Yiwuerleng | C | Hap1 | Hap1 |
| 2 | T023 | W | Hap2 | Hap1 | 6 | Ea52 | C | Hap1 | Hap1 |
| 2 | T024 | W | Hap2 | Hap1 | 6 | Buzhimeiyu | C | Hap1 | Hap1 |
| 2 | T025 | W | Hap2 | Hap3 | 6 | Haizi2hao | C | Hap2 | Hap1 |
| 2 | T028 | W | Hap2 | Hap1 | 6 | Qingyin3822 | C | Hap1 | Hap4 |
| 2 | T029 | W | Hap2 | Hap1 | 6 | 86-F021 | C | Hap3 | Hap1 |
| 2 | T031 | W | Hap1 | Hap2 | 6 | Sutanyin3hao | C | Hap3 | Hap1 |
| 2 | T032 | W | Hap1 | Hap1 | 6 | Qianjianmai | C | Hap2 | Hap1 |
| 2 | T046 | W | Hap1 | Hap2 | 6 | Wumeng6leng | C | Hap1 | Hap1 |
| 2 | T047 | W | Hap1 | Hap2 | 6 | Chunfengpidamai | C | Hap2 | Hap1 |
| 2 | T048 | W | Hap1 | Hap2 | 6 | Bizhe | C | Hap3 | Hap2 |
| 2 | T056 | W | Hap2 | Hap3 | 6 | Layiba | C | Hap3 | Hap2 |
| 2 | T062 | W | Hap1 | Hap2 | 6 | Ticn | C | Hap1 | Hap1 |
| 2 | T091 | W | Hap1 | Hap2 | 6 | Situoputexinyin10hao | C | Hap1 | Hap1 |
| 3 | T005 | W | Hap1 | Hap3 | 6 | Fengaierleng | C | Hap3 | Hap3 |
| 3 | T036 | W | Hap1 | Hap1 | 6 | Yuyaohuanghumimai | C | Hap1 | Hap1 |
| 3 | T045 | W | Hap1 | Hap1 | 7 | T026 | W | Hap1 | Hap5 |
| 3 | T059 | W | Hap1 | Hap1 | 7 | T035 | W | Hap1 | Hap3 |
| 3 | T061 | W | Hap1 | Hap1 | 7 | T037 | W | Hap1 | Hap5 |
| 3 | T064 | W | Hap1 | Hap2 | 7 | T039 | W | Hap1 | Hap1 |
| 3 | T073 | W | Hap2 | Hap2 | 7 | T040 | W | Hap2 | Hap1 |
| 3 | T074 | W | Hap1 | Hap1 | 7 | T041 | W | Hap1 | Hap1 |
| 3 | T080 | W | Hap1 | Hap1 | 7 | T042 | W | Hap1 | Hap1 |
| 3 | T085 | W | Hap1 | Hap3 | 7 | T044 | W | Hap1 | Hap2 |
| 3 | T112 | W | Hap1 | Hap1 | 7 | T051 | W | Hap1 | Hap2 |
| 4 | Mo103 | C | Hap2 | Hap1 | 7 | T053 | W | Hap1 | Hap3 |
| 4 | Nan76-31 | C | Hap1 | Hap1 | 7 | T054 | W | Hap1 | Hap2 |
| 4 | Zao3qujing | C | Hap1 | Hap1 | 7 | T057 | W | Hap1 | Hap2 |
| 4 | Zhenongbaike | C | Hap1 | Hap1 | 7 | T058 | W | Hap1 | Hap2 |
| 4 | zhahuangshen1 | C | Hap1 | Hap1 | 7 | T060 | W | Hap1 | Hap5 |
| 4 | Zhenongda5hao | C | Hap1 | Hap1 | 7 | T065 | W | Hap1 | Hap2 |
| 4 | 35-1(zhuji) | C | Hap1 | Hap1 | 7 | T067 | W | Hap1 | Hap2 |
| 4 | Zhenong12hao | C | Hap1 | Hap1 | 7 | T068 | W | Hap1 | Hap3 |
| 4 | Ribenertiao2hao | C | Hap2 | Hap1 | 7 | T069 | W | Hap1 | Hap2 |
| 4 | Riben133 | C | Hap1 | Hap1 | 7 | T071 | W | Hap1 | Hap2 |
| 4 | Gang2 | C | Hap1 | Hap1 | 7 | T072 | W | Hap1 | Hap2 |
| 4 | Meilihuangjin | C | Hap1 | Hap1 | 7 | T075 | W | Hap4 | Hap5 |
| 4 | Shang1154 | C | Hap2 | Hap1 | 7 | T076 | W | Hap1 | Hap1 |
| 4 | Clippey | C | Hap1 | Hap1 | 7 | T077 | W | Hap2 | Hap1 |
| 4 | Pisuibo | C | Hap1 | Hap1 | 7 | T078 | W | Hap2 | Hap1 |
| 4 | Wuqihuangjin | C | Hap1 | Hap2 | 7 | T079 | W | Hap1 | Hap1 |
| 4 | Feiqi5hao | C | Hap1 | Hap1 | 7 | T081 | W | Hap1 | Hap1 |
| 4 | Feiqi10hao | C | Hap2 | Hap1 | 7 | T082 | W | Hap1 | Hap3 |
| 4 | Xiu79-2 | C | Hap1 | Hap1 | 7 | T086 | W | Hap1 | Hap1 |
| 4 | Yangzhou711 | C | Hap1 | Hap1 | 7 | T087 | W | Hap1 | Hap1 |
| 4 | Sabaylis | C | Hap1 | Hap2 | 7 | T088 | W | Hap1 | Hap1 |
| 4 | Zhou87-guang4 | C | Hap1 | Hap4 | 7 | T089 | W | Hap1 | Hap1 |
| 4 | Tai91-7 | C | Hap1 | Hap1 | 7 | T090 | W | Hap1 | Hap5 |
| 4 | Zhengmingertiao | C | Hap1 | Hap1 | 7 | T093 | W | Hap1 | Hap1 |
| 4 | Guangdonghuangjin | C | Hap1 | Hap2 | 7 | T100 | W | Hap5 | Hap3 |
| 4 | Zaoshu7hao | C | Hap2 | Hap2 | 7 | T102 | W | Hap2 | Hap5 |
| 4 | Tongmai1hao | C | Hap2 | Hap4 | 7 | T107 | W | Hap2 | Hap1 |
| 4 | Dulihuang | C | Hap3 | Hap1 | 7 | T111 | W | Hap1 | Hap1 |

Note: C and W represent the cultivated barley and Tibetan wild barley, respectively.
