# Supplementary material for: Grain protein content variation and its association analysis in barley
Source: BMC Plant Biol. 2013 Mar 3;13:35. doi: 10.1186/1471-2229-13-35 (PMC3608362; doi:10.1186/1471-2229-13-35)
Supplement: Additional file 6: Figure S4 — Multiple sequence alignment of HvNAM-1 gene for different haplotypes. The symbols under the sequence alignment indicate identical residues (*), and strongly conserved (:) and weakly conserved (.) substitutions by CLUSTALW (http://align.genome.jp/). Nucleotides belong to exon are shaded in gray. The SNPs are marked in red. [file 1471-2229-13-35-S6.doc]

**Fig. S4**

DQ869678_NAM-1_ ATGGGCAGCCCGGACTCATCCTCCGGCTCGGCGCAAAAACCACCGCGGCATCAGCATCAGCATCAGCCGCCGCCTCCGCGGCGGCAGGGCTCGGCGCCGGAGCTCCCTCCCGGCTTCCGG

Hap1 ATGGGCAGCCCGGACTCATCCTCCGGCTCGGCGCAAAAACCACCGCGGCATCAGCATCAGCATCAGCCGCCGCCTCCGCGGCGGCAGGGCTCGGCGCCGGAGCTCCCTCCCGGCTTCCGG

Hap2 ATGGGCAGCCCGGACTCATCCTCCGGCTCGGCGCAAAAACCACCGCGGCATCAGCATCAGCATCAGCCGCCGCCTCCGCGGCGGCAGGGCTCGGCGCCGGAGCTCCCTCCCGGCTTCCGG

Hap3 ATGGGCAGCCCGGACTCATCCTCCGGCTCGGCGCAAAAACCACCGCGGCATCAGCATCAGCATCAGCCGCCGCCTCCGCGGCGGCAGGGCTCGGCGCCGGAGCTCCCTCCCGGCTTCCGG

Hap4 ATGGGCAGCCCGGACTCATCCTCCGGCTCGGCGCAAAAACCACCGCGGCATCAGCATCAGCATCAGCCGCCGCCTCCGCGGCGGCAGGGCTCGGCGCCGGAGCTCCCTCCCGGCTTCCGG

Hap5 ATGGGCAGCCCGGACTCATCCTCCGGCTCGGCGCAAAAACCACCGCGGCATCAGCATCAGCATCAGCCGCCGCCTCCGCGGCGGCAGGGCTCGGCGCCGGAGCTCCCTCCCGGCTTCCGG

************************************************************************************************************************

DQ869678_NAM-1_ TTCCACCCGACGGACGAGGAGCTGGTCGTGCACTACCTCAAGAAGAAGGCCGCCAAGGCGCCGCTCCCCGTCACCATCATCGCCGAGGTGGACCTCTACAAGTTCGACCCATGGGAGCTC

Hap1 TTCCACCCGACGGACGAGGAGCTGGTCGTGCACTACCTCAAGAAGAAGGCCGCCAAGGCGCCGCTCCCCGTCACCATCATCGCCGAGGTGGACCTCTACAAGTTCGACCCATGGGAGCTC

Hap2 TTCCACCCGACGGACGAGGAGCTGGTCGTGCACTACCTCAAGAAGAAGGCCGCCAAGGCGCCGCTCCCCGTCACCATCATCGCCGAGGTGGACCTCTACAAGTTCGACCCATGGGAGCTC

Hap3 TTCCACCCGACGGACGAGGAGCTGGTCGTGCACTACCTCAAGAAGAAGGCCGCCAAGGCGCCGCTCCCCGTCACCATCATCGCCGAGGTGGACCTCTACAAGTTCGACCCATG**C**GAGCTC

Hap4 TTCCACCCGACGGACGAGGAGCTGGTCGTGCACTACCTCAAGAAGAAGGCCGCCAAGGCGCCGCTCCCCGTCACCATCATCGCCGAGGTGGACCTCTACAAGTTCGACCCATGGGAGCTC

Hap5 TTCCACCCGACGGACGAGGAGCTGGTCGTGCACTACCTCAAGAAGAAGGCCGCCAAGGCGCCGCTCCCCGTCACCATCATCGCCGAGGTGGACCTCTACAAGTTCGACCCATGGGAGCTC

***************************************************************************************************************** ******

DQ869678_NAM-1_ CCCGGTATGTACTACTAGTTAGTACTATGTCTATCCCTATCTCGTCGATCGTGCTTGCTTGCTCTATCAAGCGCCGTAATTTCCCGGTGCAATTAAATAATCGAATCCGTCCACGCATCC

Hap1 CCCGGTATGTACTACTAGTTAGTACTATGTCTATCCCTATCTCGTCGATCGTGCTTGCTTGCTCTATCAAGCGCCGTAATTTCCCGGTGCAATTAAATAATCGAATCCGTCCACGCATCC

Hap2 CCCGGTATGTACTACTAGTTAGTACTATGTCTATCCCTATCTCGTCGATCGTGCTTGCTTGCTCTATCAAGCGCCGTAATTTCCCGGTGCAATTAAATAATCGAATCCGTCCACGCATCC

Hap3 CCCGGTATGTACTACTAGTTAGTACTATGTCTATCCCTATCTCGTCGATCGTGCTTGCTTGCTCTATCAAGCGCCGTAATTTCCCGGTGCAATTAAATAATCGAATCCGTCCACGCATCC

Hap4 CCCGGTATGTACTACTAGTTAGTACTATGTCTATCCCTATCTCGTCGATCGTGCTTGCTTGCTCTATCAAGCGCCGTAATTTCCCGGTGCAATTAAATAATCGAATCCGTCCACGCATCC

Hap5 CCCGGTATGTACTACTAGTTAGTACTATGTCTATCCCTATCTCGTCGATCGTGCTTGCTTGCTCTATCAAGCGCCGTAATTTCCCGGTGCAATTAAATAATCGAATCCGTCCACGCATCC

************************************************************************************************************************

DQ869678_NAM-1_ ATCCATCATGCTTTTTATTATACTGTGCACAAGTATTTTTATATTCTTCCAGTAAGTACAGCGCATGTATGTGATCCTGTCGTCGTGCTTGTTCATGCGCTCGGGCGGGATCATCATCCA

Hap1 ATCCATCATGCTTTTTATTATACTGTGCACAAGTATTTTTATATTCTTCCAGTAAGTACAGCGCATGTATGTGATCCTGTCGTCGTGCTTGTTCATGCGCTCGGGCGGGATCATCATCCA

Hap2 ATCCATCATGCTTTTTATTATACTGTGCACAAGTATTTTTATATTCTTCCAGTAAGTACAGCGCATGTATGTGATCCTGTCGTCGTGCTTGTTCATGCGCTCGGGCGGGATCATCATCCA

Hap3 ATCCATCATGCTTTTTATTATACTGTGCACAAGTATTTTTATATTCTTCCAGTAAGTACAGCGCATGTATGTGATCCTGTCGTCGTGCTTGTTCATGCGCTCGGGCGGGATCATCATCCA

Hap4 ATCCATCATGCTTTTTATTATACTGTGCACAAGTATTTTTATATTCTTCCAGTAAGTACAGCGCATGTATGTGATCCTGTCGTCGTGCTTGTTCATGCGCTCGGGCGGGATCATCATCCA

Hap5 ATCCATCATGCTTTTTATTATACTGTGCACAAGTATTTTTATATTCTTCCAGTAAGTACAGCGCATGTATGTGATCCTGTCGTCGTGCTTGTTCATGCGCTCGGGCGGGATCATCATCCA

************************************************************************************************************************

DQ869678_NAM-1_ TCAGAGAAGGCGACCTTCGGGGAGCACGAGTGGTACTTCTTCAGCCCGCGCGACCGCAAGTACGCCAACGGCGCGCGGCCGAACCGGGCGGCGACGTCGGGCTACTGGAAGGCCACCGGC

Hap1 TCAGAGAAGGCGACCTTCGGGGAGCACGAGTGGTACTTCTTCAGCCCGCGCGACCGCAAGTACGCCAACGGCGCGCGGCCGAACCGGGCGGCGACGTCGGGCTACTGGAAGGCCACCGGC

Hap2 TCAGAGAAGGCGACCTTCGGGGAGCACGAGTGGTACTTCTTCAGCCCGCGCGACCGCAAGTACCCCAACGGCGCGCGGCCGAACCGGGCGGCGACGTCGGGCTACTGGAAGGCCACCGGC

Hap3 TCAGAGAAGGCGACCTTCGGGGAGCACGAGTGGTACTTCTTCAGCCCGCGCGACCGCAAGTACCCCAACGGCGCGCGGCCGAACCGGGCGGCGACGTCGGGCTACTGGAAGGCCACCGGC

Hap4 TCAGAGAAGGCGACCTTCGGGGAGCACGAGTGGTACTTCTTCAGCCCGCGCGACCGCAAGTACCCCAACGGCGCGCGGCCGAACCGGGCGGCGACGTCGGGCTACTGGAAGGCCACCGGC

Hap5 TCAGAGAAGGCGACCTTCGGGGAGCACGAGTGGTACTTCTTCAGCCCGCGCGACCGCAAGTACCCCAACGGCGCGCGGCCGAACCGGGCGGCGACGTCGGGCTACTGGAAGGCCACCGGC

*************************************************************** ********************************************************

DQ869678_NAM-1_ ACGGACAAGCCTATCCTGGCCTCGGCCACCGGGTGCGGCCGGGAGAAGGTCGGCGTCAAGAAGGCGCTCGTCTTCTACCGCGGGAAGCCGCCCAGGGGCCTCAAGACCAACTGGATCATG

Hap1 ACGGACAAGCCTATCCTGGCCTCGGCCACCGGGTGCGGCCGGGAGAAGGTCGGCGTCAAGAAGGCGCTCGTCTTCTACCGCGGGAAGCCGCCCAGGGGCCTCAAGACCAACTGGATCATG

Hap2 ACGGACAAGCCTATCCTGGCCTCGGCCACCGGGTGCGGCCGGGAGAAGGTCGGCGTCAAGAAGGCGCTCGTCTTCTACCGCGGGAAGCCGCCCAGGGGCCTCAAGACCAACTGGATCATG

Hap3 ACGGACAAGCCTATCCTGGCCTCGGCCACCGGGTGCGGCCGGGAGAAGGTCGGCGTCAAGAAGGCGCTCGTCTTCTACCGCGGGAAGCCGCCCAGGGGCCTCAAGACCAACTGGATCATG

Hap4 ACGGACAAGCCTATCCTGGCCTCGGCCACCGGGTGCGGCCGGGAGAAGGTCGGCGTCAAGAAGGCGCTCGTCTTCTACCGCGGGAAGCCGCCCAGGGGCCTCAAGACCAACTGGATCATG

Hap5 ACGGACAAGCCTATCCTGGCCTCGGCCACCGGGTGCGGCCGGGAGAAGGTCGGCGTCAAGAAGGCGCTCGTCTTCTACCGCGGGAAGCCGCCCAGGGGCCTCAAGACCAACTGGATCATG

************************************************************************************************************************

DQ869678_NAM-1_ CATGAGTACCGCCTCACCGGAGCCTCTGCTGGCTCCACCACCACCAGCCGGCCGCCGCCGGTGACCGGCGGGAGCAGGGCCCCGGCCTCTCTCAGGGTACGTACTTACACGTGTCCATCG

Hap1 CATGAGTACCGCCTCACCGGAGCCTCTGCTGGCTCCACCACCACCAGCCGGCCGCCGCCGGTGACCGGCGGGAGCAGGGCCCCGGCCTCTCTCAGGGTACGTACTTACACGTGTCCATCG

Hap2 CATGAGTACCGCCTCACCGGAGCCTCTGCTGGCTCCACCACCACCAGCCGGCCGCCGCCGGTGACCGGCGGGAGCAGGGCCCCGGCCTCTCTCAGGGTACGTACTTACACGTGTCCATCG

Hap3 CATGAGTACCGCCTCACCGGAGCCTCTGCTGGCTCCACCACCACCAGCCGGCCGCCGCCGGTGACCGGCGGGAGCAGGGCCCCGGCCTCTCTCAGGGTACGTACTTACACGTGTCCATCG

Hap4 CATGAGTACCGCCTCACCGGAGCCTCTGCTGGCTCCACCACCACCAGCCGGCCGCCGCCGGTGACCGGCGGGAGCAGGGCCCCGGCCTCTCTCAGGGTACGTACTTACACGTGTCCATCG

Hap5 CATGAGTACCGCCTCACCGGAGCCTCTGCTGGCTCCACCACCACCAGCCGGCCGCCGCCGGTGACCGGCGGGAGCAGGGCCCCGGCCTCTCTCAGGGTACGTACTTACACGTGTCCATCG

************************************************************************************************************************

DQ869678_NAM-1_ CACGGTCTATCAGTATTTATTTATTAACTACTCTCGAGCTTAATTATGGTATTGTTGATAGTTGATGAAGTTAATTATTGTACGCCGTCTCATCGATCAGTTGGACGACTGGGTGCTGTG

Hap1 CACGGTCTATCAGTATTTATTTATTAACTACTCTCGAGCTTAATTATGGTATTGTTGATAGTTGATGAAGTTAATTATTGTACGCCGTCTCATCGATCAGTTGGACGACTGGGTGCTGTG

Hap2 CACGGTCTATCAGTATTTATTTATTAACTACTCTCGAGCTTAATTATGGTATTGTTGATAGTTGATGAAGTTAATTATTGTACGCCGTCTCATCGATCAGTTGGACGACTGGGTGCTGTG

Hap3 CACGGTCTATCAGTATTTATTTATTAACTACTCTCGAGCTTAATTATGGTATTGTTGATAGTTGATGAAGTTAATTATTGTACGCCGTCTCATCGATCAGTTGGACGACTGGGTGCTGTG

Hap4 CACGGTCTATCAGTATTTATTTATTAACTACTCTCGAGCTTAATTATGGTATTGTTGATAGTTGATGAAGTTAATTATTGTACGCCGTCTCATCGATCAGTTGGACGACTGGGTGCTGTG

Hap5 CACGGTCTATCAGTATTTATTTATTAACTACTCTCGAGCTTAATTATGGTATTGTTGATAGTTGATGAAGTTAATTATTGTACGCCGTCTCATCGATCAGTTGGACGACTGGGTGCTGTG

************************************************************************************************************************

DQ869678_NAM-1_ CCGCATCTACAAGAAGACCAGCAAGGCCGCGGCCGCGGTCGGAGATGAGCAGAGGAGCATGGAGTGCGAGGACTCCGTGGAGGACGCGGTCACCGCGTACCCGCCCTACGCCACGGCGGG

Hap1 CCGCATCTACAAGAAGACCAGCAAGGCCGCGGCCGCGGTCGGAGATGAGCAGAGGAGCATGGAGTGCGAGGACTCCGTGGAGGACGCGGTCACCGCGTACCCGCCCTACGCCACGGCGGG

Hap2 CCGCATCTACAAGAAGACCAGCAAGGCCGCGGCCGCGGTCGGAGATGAGCAGAGGAGCATGGAGTGCGAGGACTCCGTGGAGGACGCGGTCACCGCGTACCCGCCCTACGCCACGGCGGG

Hap3 CCGCATCTACAAGAAGACCAGCAAGGCCGCGGCCGCGGTCGGAGATGAGCAGAGGAGCATGGAGTGCGAGGACTCCGTGGAGGACGCGGTCACCGCGTACCCGCCCTACGCCACGGCGGG

Hap4 CCGCATCTACAAGAAGACCAGCAAGGCCGCGGCCGCGGTCGGAGATGAGCAGAGGAGCATGGAGTGCGAGGACTCCGTGGAGGACGCGGTCACCGCGTACCCGCCCTACGCCACGGCGGG

Hap5 CCGCATCTACAAGAAGACCAGCAAGGCCGCGGCCGCGGTCGGAGATGAGCAGAGGAGCATGGAGTGCGAGGACTCCGTGGAGGACGCGGTCACCGCGTACCCGCCCTACGCCACGGCGGG

************************************************************************************************************************

DQ869678_NAM-1_ CATGGCCGGCGCAGGTGCGCATGGCAGCAACTACGTTCAACTGCTCCATCATCACGACAGCCACGAGGACAACTTCCAGCTAGACGGCCTGCTCACAGAACACGACGTCGGCCTCTCGGC

Hap1 CATGGCCGGCGCAGGTGCGCATGGCAGCAACTACGTTCAACTGCTCCATCATCACGACAGCCACGAGGACAACTTCCAGCTAGACGGCCTGCTCACAGAACACGACGTCGGCCTCTCGGC

Hap2 CATGGCCGGCGCAGGTGCGCATGGCAGCAACTACGTTCAACTGCTCCATCATCACGACAGCCACGAGGACAACTTCCAGCTAGACGGCCTGCTCACAGAACACGACGTCGGCCTCTCGGC

Hap3 CATGGCCGGCGCAGGTGCGCATGGCAGCAACTACGTTCAACTGCTCCATCATCACGACAGCCACGAGGACAACTTCCAGCTAGACGGCCTGCTCACAGAACACGACGTCGGCCTCTCGGC

Hap4 CATGGCCGGCGCAGGTGCGCATGGCAGCAACTACGTTCAACTGCTCCATCATCACGACAGCCACGAGGACAACTTCCAGCTAGACGGCCTGCTCACAGAACACGACGTCAGCCTCTCGGC

Hap5 CATGGCCGGCGCAGGTGCGCATGGCAGCAACTACGTTCAACTGCTCCATCATCACGACAGCCACGAGGACAACTTCCAGCTAGACGGCCTGCTCACAGAACACGACGTCAGCCTCTCGGC

************************************************************************************************************* **********

DQ869678_NAM-1_ GGGCGCCGCCTCGCTGGGCCACCTTGCCGCGGCGGCGAGGGCCACCAAACAGTTCCTCGCCCCGTCGTCCTCAACCCCGTTCAACTGGCTCGAGGCGTCAACCGGTGGCAGCATCCTCCC

Hap1 GGGCGCCGCCTCGCTGGGCCACCTTGCCGCGGCGGCGAGGGCCACCAAACAGTTCCTCGCCCCGTCGTCCTCAACCCCGTTCAACTGGCTCGAGGCGTCAACCGGTGGCAGCATCCTCCC

Hap2 GGGCGCCGCCTCGCTGGGCCACCTTGCCGCGGCGGCGAGGGCCACCAAACAGTTCCTCGCCCCGTCGTCCTCAACCCCGTTCAACTGGCTCGAGGCGTCAACCGGTGGCAGCATCCTCCC

Hap3 GGGCGCCGCCTCGCTGGGCCACCTTGCCGCGGCGGCGAGGGCCACCAAACAGTTCCTCGCCCCGTCGTCCTCAACCCCGTTCAACTGGCTCGAGGCGTCAACCGGTGGCAGCATCCTCCC

Hap4 GGGCGCCGCCTCGCTGGGCCACCTTGCCGCGGCGGCGAGGGCCACCAAACAGTTCCTCGCCCCGTCGTCCTCAACCCCGTTCAACTGGCTCGAGGCGTCAACCGGTGGCAGCATCCTCCC

Hap5 GGGCGCCGCCTCGCTGGGCCACCTTGCCGCGGCGGCGAGGGCCACCAAACAGTTCCTCGCCCCGTCGTCCTCAACCCCGTTCAACTGGCTCGAGGCGTCAACCGGTGGCAGCATCCTCCC

************************************************************************************************************************

DQ869678_NAM-1_ ACAGGCAAGGAATTTCCCTGGGTTTAACAGGAGCAGAAACGTCGGCAGTATGTCGCTGTCATCCACGGCCGACGACATGGCTGGCGCGGTGGACGTCAGCGACGGAGGCAATGCGGTGAA

Hap1 ACAGGCAAGGAATTTCCCTGGGTTTAACAGGAGCAGAAACGTCGGCAGTATGTCGCTGTCATCCACGGCCGACGACATGGCTGGCGCGGTGGACGTCAGCGACGGAGGCAATGCGGTGAA

Hap2 ACAGGCAAGGAATTTCCCTGGGTTTAACAGGAGCAGAAACGTCGGCAGTATGTCGCTGTCATCCACGGCCGACGACATGGCTGGCGCGGTGGACGTCAGCGACGGAGGCAATGCGGTGAA

Hap3 ACAGGCAAGGAATTTCCCTGGGTTTAACAGGAGCAGAAACGTCGGCAGTATGTCGCTGTCATCCACGGCCGACGACATGGCTGGCGCGGTGGACGTCAGCGACGGAGGCAATACGGTGAA

Hap4 ACAGGCAAGGAATTTCCCTGGGTTTAACAGGAGCAGAAACGTCGGCAGTATGTCGCTGTCATCCACGGCCGACGACATGGCTGGCGCGGTGGACGTCAGCGACGGAGGCAATGCGGTGAA

Hap5 ACAGGCAAGGAATTTCCCTGGGTTTAACAGGAGCAGAAACGTCGGCAGTATGTCGCTGTCATCCACGGCCGACGACATGGCTGGCGCGGTGGACGTCAGCGACGGACGCAATGCGGTGAA

********************************************************************************************************** ***** *******

DQ869678_NAM-1_ CGCCATGTATCTCCCCGTGCAAGACGGGACCTACCATCAGCATGTCATCCTCGGAGCTCCGCTGGCGCCAGAGGCCATCGCGGGCGCCGCCACCTCTGGTTTCCAGCATCACGTCCAAAT

Hap1 CGCCATGTATCTCCCCGTGCAAGACGGGACCTACCATCAGCATGTCATCCTCGGAGCTCCGCTGGCGCCAGAGGCCATCGCGGGCGCCGCCACCTCTGGTTTCCAGCATCACGTCCAAAT

Hap2 CGCCATGTATCTCCCCGTGCAAGACGGGACCTACCATCAGCATGTCATCCTCGGAGCTCCGCTGGCGCCAGAGGCCATCGCGGGCGCCGCCACCTCTGGTTTCCAGCATCACGTCCAAAT

Hap3 CGCCATGTATCTCCCCGTGCAAGACGGGACCTACCATCAGCATGTCATCCTCGGAGCTCCGCTGGCGCCAGAGGCCATCGCGGGCGCCGCCACCTCTGGTTTCCAGCATCACGTCCAAAT

Hap4 CGCCATGTATCTCCCCGTGCAAGACGGGACCTACCATCAGCATGTCATCCTCGGAGCTCCGCTGGCGCCAGAGGCCATCGCGGGCGCCGCCACCTCTGGTTTCCAGCATCACGTCCAAAT

Hap5 CGCCATGTATCTCCCCGTGCAAGACGGGACCTACCATCAGCATGTCATCCTCGGAGCTCCGCTGGCGCCAGAGGCCATCGCGGGCGCCGCCACCTCTGGTTTCCAGCATCACGTCCAAAT

************************************************************************************************************************

DQ869678_NAM-1_ ATCCGGCGTGAACTGGAATCCCTGA

Hap1 ATCCGGCGTGAACTGGAATCCCTGA

Hap2 ATCCGGCGTGAACTGGAATCCCTGA

Hap3 ATCCGGCGTGAACTGGAATCCCTGA

Hap4 ATCCGGCGTGAACTGGAATCCCTGA

Hap5 ATCCGGCGTGAACTGGAATCCCTGA

*************************
