# Supplementary material for: Grain protein content variation and its association analysis in barley
Source: BMC Plant Biol. 2013 Mar 3;13:35. doi: 10.1186/1471-2229-13-35 (PMC3608362; doi:10.1186/1471-2229-13-35)
Supplement: Additional file 7: Figure S5 — Multiple sequence alignment of HvNAM-2 gene for different haplotypes. The symbols under the sequence alignment indicate identical residues (*), and strongly conserved (:) and weakly conserved (.) substitutions by CLUSTALW (http://align.genome.jp/). Nucleotides belong to exon are shaded in gray. The SNPs are marked in red. [file 1471-2229-13-35-S7.doc]

**Fig. S5**

DQ869679_NAM-2_ ATGGGCAGCTCGGACTCATCTTCCGGCTCGGCACCGCCGCGGCATCAGCCGCCGCCTCCGCAGCAGGGCTCGGCGCCGGAGCTCCCGCCGGGCTTCCGGTTCCACCCCACAGACGAGGAG

Hap1 ATGGGCAGCTCGGACTCATCTTCCGGCTCGGCACCGCCGCGGCATCAGCCGCCGCCTCCGCAGCAGGGCTCGGCGCCGGAGCTCCCGCCGGGCTTCCGGTTCCACCCCACAGACGAGGAG

Hap2 ATGGGCAGCTCGGACTCATCTTCCGGCTCGGCACCGCCGCGGCATCAGCCGCCGCCTCCGCAGCAGGGCTCGGCGCCGGAGCTCCCGCCGGGCTTCCGGTTCCACCCCACAGACGAGGAG

Hap3 ATGGGCAGCTCGGACTCATCTTCCGGCTCGGCACCGCCGCGGCATCAGCCGCCGCCTCCGCAGCAGGGCTCGGCGCCGGAGCTCCCGCCGGGCTTCCGGTTCCACCCCACAGACGAGGAG

Hap4 ATGGGCAGCTCGGACTCATCTTCCGGCTCGGCACCGCCGCGGCATCAGCCGCCGCCTCCGCAGCAGGGCTCGGCGCCGGAGCTCCCGCCGGGCTTCCGGTTCCACCCCACAGACGAGGAG

Hap5 ATGGGCAGCTCGGACTCATCTTCCGGCTCGGCACCGCCGCGGCATCAGCCGCCGCCTCCGCAGCAGGGCTCGGCGCCGGAGCTCCCGCCGGGCTTCCGGTTCCACCCCACAGACGAGGAG

Hap6 ATGGGCAGCTCGGACTCATCTTCCGGCTCGGCACCGCCGCGGCATCAGCCGCCGCCTCCGCAGCAGGGCTCGGCGCCGGAGCTCCCGCCGGGCTTCCGGTTCCACCCCACAGACGAGGAG

************************************************************************************************************************

DQ869679_NAM-2_ CTGGTCGTGCACTACCTCAAGAAGAAGGCCGCCAAGGTGCCGCTCCCTGTCACCATCATCGCCGAGGTGGATCTCTACAAGTTCGACCCATGGGAGCTCCCCGGTACGTATGTATGTAAA

Hap1 CTGGTCGTGCACTACCTCAAGAAGAAGGCCGCCAAGGTGCCGCTCCCTGTCACCATCATCGCCGAGGTGGATCTCTACAAGTTCGACCCATGGGAGCTCCCCGGTACGTATGTATGTAAA

Hap2 CTGGTCGTGCACTACCTCAAGAAGAAGGCCGCCAAGGTGCCGCTCCCTGTCACCATCATCGCCGAGGTGGATCTCTACAAGTTCGACCCATGGGAGCTCCCCGGTACGTATGTATGTAAA

Hap3 CTGGTCGTGCACTACCTCAAGAAGAAGGCCGCCAAGGTGCCGCTCCCTGTCACCATCATCGCCGAGGTGGATCTCTACAAGTTCGACCCATGGGAGCTCCCCGGTACGTATGTATGTAAA

Hap4 CTGGTCGTGCACTACCTCAAGAAGAAGGCCGCCAAGGTGCCGCTCCCTGTCACCATCATCGCCGAGGTGGATCTCTACAAGTTCGACCCATGGGAGCTCCCCGGTACGTATGTATGTAAA

Hap5 CTGGTCGTGCACTACCTCAAGAAGAAGGCCGCCAAGGTGCCGCTCCCTGTCACCATCATCGCCGAGGTGGATCTCTACAAGTTCGACCCATGGGAGCTCCCCGGTACGTATGTATGTAAA

Hap6 CTGGTCGTGCACTACCTCAAGAAGAAGGCCGCCAAGGTGCCGCTCCCTGTCACCATCATCGCCGAGGTGGATCTCTACAAGTTCGACCCATGGGAGCTCCCCGGTACGTATGTATGTAAA

************************************************************************************************************************

DQ869679_NAM-2_ TATCTCGTCGTGCTTATCAAGCGCCGTAAATTTTCCGGTGCAATTAAATAATAGATCGAATCCATCGATCATGCTTGTACACTACTGTGCAAGAAGTATTTTTATATTGTTTCAGTACAC

Hap1 TATCTCGTCGTGCTTATCAAGCGCCGTAAATTTTCCGGTGCAATTAAATAATAGATCGAATCCATCCATCATGCTTGTACACTACTGTGCAAGAAGTATTTTTATATTGTTTCAGTACAC

Hap2 TATCTCGTCGTGCTTATCAAGCGCCGTAAATTTTCCGGTGCAATTAAATAATAGATCGAATCCATCCATCATGCTTGTACACTACTGTGCAAGAAGTATTTTTATATTGTTTCAGTACAC

Hap3 TATCTCGTCGTGCTTATCAAGCGCCGTAAATTTTCCGGTGCAATTAAATAATAGATCGAATCCATCGATCATGCTTGTACACTACTGTGCAAGAAGTATTTTTATATTGTTTCAGTACAC

Hap4 TATCTCGTCGTGCTTATCAAGCGCCGTAAATTTTCCGGTGCAATTAAATAATAGATCGAATCCATCGATCATGCTTGTACACTACTGTGCAAGAAGTATTTTTATATTGTTTCAGTACAC

Hap5 TATCTCGTCGTGCTTATCAAGCGCCGTAAATTTTCCGGTGCAATTAAATAATAGATCGAATCCATCGATCATGCTTGTACACTACTGTGCAAGAAGTATTTTTATATTGTTTCAGTACAC

Hap6 TATCTCGTCGTGCTTATCAAGCGCCGTAAATTTTCCGGTGCAATTAAATAATAGATCGAATCCATCCATCATGCTTGTACACTACTGTGCAAGAAGTATTTTTATATTGTTTCAGTACAC

****************************************************************** *****************************************************

DQ869679_NAM-2_ ATGTATGTAGATGGTTTATGTATGTGATCCTGTCGTGCTTGTTCATGCGCTCGCTCGGGATCGATCAGAGAAGGCGACCTTCGGGGAGCAGGAGTGGTACTTCTTCAGCCCGCGCGACCG

Hap1 ATGTATGTAGATGGTTTATGTATGTGATCCTGTCGTGCTTGTTCATGCGCTCGCTCGGGATCGATCAGAGAAGGCGACCTTCGGGGAGCAGGAGTGGTACTTCTTCAGCCCGCGCGACCG

Hap2 ATGTATGTAGATGGTTTATGTATGTGATCCTGTCGTGCTTGTTCATGCGCTCGCTCGGGATCGATCAGAGAAGGCGACCTTCGGGGAGCAGGAGTGGTACTTCTTCAGCCCGCGCGACCG

Hap3 ATGTATGTAGATGGTTTATGTATGTGATCCTGTCGTGCTTGTTCATGCGCTCGCTCGGGATCGATCAGAGAAGGCGACCTTCGGGGAGCAGGAGTGGTACTTCTTCAGCCCGCGCGACCG

Hap4 ATGTATGTAGATGGTTTATGTATGTGATCCTGTCGTGCTTGTTCATGCGCTCGCTCGGGATCGATCAGAGAAGGCGACCTTCGGGGAGCAGGAGTGGTACTTCTTCAGCCCGCGCGACCG

Hap5 ATGTATGTAGATGGTTTATGTATGTGATCCTGTCGTGCTTGTTCATGCGCTCGCTCGGGATCGATCAGAGAAGGCGACCTTCGGGGAGCAGGAGTGGTACTTCTTCAGCCCGCGCGACCG

Hap6 ATGTATGTAGATGGTTTATGTATGTGATCCTGTCGTGCTTGTTCATGCGCTCGCTCGGGATCGATCAGAGAAGGCGACCTTCGGGGAGCAGGAGTGGTACTTCTTCAGCCCGCGCGACCG

************************************************************************************************************************

DQ869679_NAM-2_ CAAGTATCCCAACGGCGCGCGGCCCAACAGGGCGGCCACGTCGGGGTACTGGAAGGCGACCGGCACGGACAAGCCCATCCTGGCCTCTGGGTGCGGCCGGGAGAAGGTCGGCGTCAAGAA

Hap1 CAAGTATCCCAACGGCGCGCGGCCCAACAGGGCGGCCACGTCGGGGTACTGGAAGGCGACCGGCACGGACAAGCCCATCCTGGCCTCTGGGTGCGGCCGGGAGAAGGTCGGCGTCAAGAA

Hap2 CAAGTATCCCAACGGCGCGCGGCCCAACAGGGCGGCCACGTCGGGGTACTGGAAGGCGACCGGCACGGACAAGCCCATCCTGGCCTCTGGGTGCGGCCGGGAGAAGGTCGGCGTCAAGAA

Hap3 CAAGTATCCCAACGGCGCGCGGCCCAACAGGGCGGCCACGTCGGGGTACTGGAAGGCGACCGGCACGGACAAGCCCATCCTGGCCTCTGGGTGCGGCCGGGAGAAGGTCGGCGTCAAGAA

Hap4 CAAGTATCCCAACGGCGCGCGGCCCAACAGGGCGGCCACGTCGGGGTACTGGAAGGCGACCGGCACGGACAAGCCCATCCTGGCCTCTGGGTGCGGCCGGGAGAAGGTCGGCGTCAAGAA

Hap5 CAAGTATCCCAACGGCGCGCGGCCCAACAGGGCGGCCACGTCGGGGTACTGGAAGGCGACCGGCACGGACAAGCCCATCCTGGCCTCTGGGTGCGGCCGGGAGAAGGTCGGCGTCAAGAA

Hap6 CAAGTATCCCAACGGCGCGCGGCCCAACAGGGCGGCCACGTCGGGGTACTGGAAGGCGACCGGCACGGACAAGCCCATCCTGGCCTCTGGGTGCGGCCGGGAGAAGGTCGGCGTCAAGAA

************************************************************************************************************************

DQ869679_NAM-2_ NGCGCTCGTCTTCTACCGCGGGAAGCCGCCCAAGGGCCTCAAAACCAACTGGATCATGCACGAGTACCGCCTCACCGACGCGTCTAGCTCCGCCGCCACCAGCCGACCTCCGCCCGTGAC

Hap1 NGCGCTCGTCTTCTACCGCGGGAAGCCGCCCAAGGGCCTCAAAACCAACTGGATCATGCACGAGTACCGCCTCACCGACGCGTCTAGCTCCGCCGCCACCAGCCGACCTCCGCCCGTGAC

Hap2 NGCGCTCGTCTTCTACCGCGGGAAGCCGCCCAAGGGCCTCAAAACCAACTGGATCATGCACGAGTACCGCCTCACCGACGCGTCTAGCTCCGCCGCCACCAGCCGACCTCCGCCCGTGAC

Hap3 GGCGCTCGTCTTCTACCGCGGGAAGCCGCCCAAGGGCCTCAAAACCAACTGGATCATGCACGAGTACCGCCTCACCGACGCGTCTAGCTCCGCCGCCACCAGCCGACCTCCGCCCGTGAC

Hap4 NGCGCTCGTCTTCTACCGCGGGAAGCCGCCCAAGGGCCTCAAAACCAACTGGATCATGCACGAGTACCGCCTCACCGACGCGTCTAGCTCCGCCGCCACCAGCCGACCTCCGCCCGTGAC

Hap5 NGCGCTCGTCTTCTACCGCGGGAAGCCGCCCAAGGGCCTCAAAACCAACTGGATCATGCACGAGTACCGCCTCACCGACGCGTCTAGCTCCGCCGCCACCAGCCGACCTCCGCCCGTGAC

Hap6 NGCGCTCGTCTTCTACCGCGGGAAGCCGCCCAAGGGCCTCAAAACCAACTGGATCATGCACGAGTACCGCCTCACCGACGCGTCTAGCTCCGCCGCCACCAGCCGACCTCCGCCCGTGAC

***********************************************************************************************************************

DQ869679_NAM-2_ CGGAGGGAGCAGGGCTGCCTCTCTCAGGGTACGTGTCGACCGATCGCACGGTCAAGCAGTAACCGATCTCCGTATTTCAGTACTATATCGAGCTTAGGGTATTGTGGTTGATGAAGTTAA

Hap1 CGGAGGGAGCAGGGCTGCCTCTCTCAGGGTACGTGTCGACCGATCGCACGGTCAAGCAGTAACCGATCTCCGTATTTAAGTACTATATCGAGCTTAGGGTATTGTGGTTGATGAAGTTAA

Hap2 CGGAGGGAGCAGGGCTGCCTCTCTCAGGGTACGTGTCGACCGATCGCACGGTCAAGCAGTAACCGATCTCCGTATTTCAGTACTATATCGAGCTTAGGGTATTGTGGTTGATGAAGTTAA

Hap3 CGGAGGGAGCAGGGCTGCCTCTCTCAGGGTACGTGTCGACCGATCGCACGGTCAAGCAGTAACCGATCTCCGTATTTCAGTACTATATCGAGCTTAGGGTATTGTGGTTGATGAAGTTAA

Hap4 CGGAGGGAGCAGGGCTGCCTCTCTCAGGGTACGTGTCGACCGATCGCACGGTCAAGCAGTAACCGATCTCCGTATTTCAGTACTATATCGAGCTTAGGGTATTGTGGTTGATGAAGTTAA

Hap5 CGGAGGGAGCAGGGCTGCCTCTCTCAGGGTACGTGTCGACCGATCGCACGGTCAAGCAGTAACCGATCTCCGTATTTAAGTACTATATCGAGCTTAGGGTATTGTGGTTGATGAAGTTAA

Hap6 CGGAGGGAGCAAGGCTGCCTCTCTCAGGGTACGTGTCGACCGATCGCACGGTCAAGCAGTAACCGATCTCCGTATTTAAGTACTATATCGAGCTTAGGGTATTGTGGTTGATGAAGTTAA

*********** ***************************************************************** ******************************************

DQ869679_NAM-2_ TTGGTGCACGTCGTCTCACCAGTTGGATGACTGGGTGCTGTGCCGCATATACAAGAAGATCAACAAGGCCGCCGCCGCGGATCAGCAGAGGAGCATGGAGTGCGAGGACTCCGTGGAGGA

Hap1 TTGGTGCACGTCGTCTCACCAGTTGGATGACTGGGTGCTGTGCCGCATATACAAGAAGATCAACAAGGCCGCCGCCGCGGATCAGCAGAGGAGCATGGAGTGCGAGGACTCCGTGGAGGA

Hap2 TTGGTGCACGTCGTCTCACCAGTTGGATGACTGGGTGCTGTGCCGCATATACAAGAAGATCAACAAGGCCGCCGCCGCGGATCAGCAGAGGAGCATGGAGTGCGAGGACTCCGTGGAGGA

Hap3 TTGGTGCACGTCGTCTCACCAGTTGGATGACTGGGTGCTGTGCCGCATATACAAGAAGATCAACAAGGCCGCCGCCGCGGATCAGCAGAGGAGCATGGAGTGCGAGGACTCCGTGGAGGA

Hap4 TTGGTGCACGTCGTCTCACCAGTTGGATGACTGGGTGCTGTGCCGCATATACAAGAAGATCAACAAGGCCGCCGCCGCGGATCAGCAGAGGAGCATGGAGTGCGAGGACTCCGTGGAGGA

Hap5 TTGGTGCACGTCGTCTCACCAGTTGGATGACTGGGTGCTGTGCCGCATATACAAGAAGATCAACAAGGCCGCCGCCGCGGATCAGCAGAGGAGCATGGAGTGCGAGGACTCCGTGGAGGA

Hap6 TTGGTGCACGTCGTCTCACCAGTTGGATGACTGGGTGCTGTGCCGCATATACAAGAAGATCAACAAGGCCGCCGCCGCGGATCAGCAGAGGAGCATGGAGTGCGAGGACTCCGTGGAGGA

************************************************************************************************************************

DQ869679_NAM-2_ CGCCGTCACCGCATACCCGCCGTATGCCACAGCGTGCATGACCGGTGAAGGGGCGCACGGCAGCAACTACGCTTCACTGCTCCATCACCAGGACAGCCACGAGGACAACTTCCTGGACGG

Hap1 CGCCGTCACCGCATACCCTCCGTATGCCACGGCGTGCATGACCGGTGAAGGGGCGCACGGCAGCAACTACGCTTCACTGCTCCATCACCAGGACAGCCACGAGGACAACTTCCTGGACGG

Hap2 CGCCGTCACCGCATACCCTCCGTATGCCACGGCGTGCATGACCGGTGAAGGGGCGCACGGCAGCAACTACGCTTCACTGCTCCATCACCAGGACAGCCACGAGGACAACTTCCTGGACGG

Hap3 CGCCGTCACCGCATACCCGCCGTATGCCACAGCGTGCATGACCGGTGAAGGGGCGCACGGCAGCAACTACGCTTCACTGCTCCATCACCAGGACAGCCACGAGGACAACTTCCTGGACGG

Hap4 CGCCGTCACCGCATACCCGCCGTATGCCACGGCGTGCATGACCGGTGAAGGGGCGCACGGCAGCAACTACGCTTCACTGCTCCATCACCAGGACAGCCACGAGGACAACTTCCTGGACGG

Hap5 CGCCGTCACCGCATACCCGCCGTATGCCACGGCGTGCATGACCGGTGAAGGGGCGCACGGCAGCAACTACGCTTCACTGCTCCATCACCAGGACAGCCACGAGGACAACTTCCTGGACGG

Hap6 CACCGTCACCGCATACCCGCCGTATGCCACGGCGTGCATGACCGGTGAAGGGGCGCACGGCAGCAACTACGCTGCACTGCTCCATCACCAGGACAGCCACGAGGACAACTTCCTGGACGG

* **************** *********** ****************************************** **********************************************

DQ869679_NAM-2_ CCTGCTCACAGCAGAGGACGCCGGACTCTCGGCGGGCGCCACCTCGCTGAGCCACCTAGCCGCGGCGGCGAGGGGGAGCCCGGCTCCGACCAAACAGTTTCTCGCCCCGTCATCGTCAAC

Hap1 CCTGCTCACAGCAGAGGACGCCGGACTCTCGGCGGGCGCCACCTCGCTGAGCCACCTAGCCGCGGCGGCGAGGGGGAGCCCGGCTCCGACCAAACAGTTTCTCGCCCCGTCATCGTCAAC

Hap2 CCTGCTCACAGCAGAGGACGCCGGACTCTCGGCGGGCGCCACCTCGCTGAGCCACCTAGCCGCGGCGGCGAGGGGGAGCCCGGCTCCGACCAAACAGTTTCTCGCCCCGTCATCGTCAAC

Hap3 CCTGCTCACAGCAGAGGACGCCGGACTCTCGGCGGGCGCCACCTCGCTGAGCCACCTAGCCGCGGCGGCGAGGGGGAGCCCGGCTCCGACCAAACAGTTTCTCGCCCCGTCATCGTCAAC

Hap4 CCTGCTCACAGCAGAGGACGCCGGACTCTCGGCGGGCGCCACCTCGCTGAGCCACCTAGCCGCGGCGGCGAGGGGGAGCCCGGCTCCGACCAAACAGTTTCTCGCCCCGTCATCGTCAAC

Hap5 CCTGCTCACAGCAGAGGACGCCGGACTCTCGGCGGGCGCCACCTCGCTGAGCCACCTAGCCGCGGCGGCGAGGGGGAGCCCGGCTCCGACCAAACAGTTTCTCGCCCCGTCATCGTCAAC

Hap6 CCTGCTCACAGCAGAGGACGCCGGACTCTCGGCGGGCGCCACCTCGCTGAGCCACCTAGCCGCGGCGGCGAGGGGGAGCCCGGCTCCGACCAAACAGTTTCTCGCCCCGTCATCGTCAAC

************************************************************************************************************************

DQ869679_NAM-2_ CCAATTCAACTGGCTCGATGCGTCAACCGTTGGCATCCTCCCACATGCAAGGAATTTTCCTGGGTTTAACAGGAGCAGAAACGTCGGAAATATGTCGCTGTCATCGACGGCCGACATGGC

Hap1 CCAATTCAACTGGCTCGATGCGTCAACCGTTGGCATCCTCCCACATGCAAGGAATTTTCCTGGGTTTAACAGGAGCAGAAACGTCGGAAATATGTCGCTGTCATCGACGGCCGACATGGC

Hap2 CCAATTCAACTGGCTCGATGCGTCAACCGTTGGCATCCTCCCACATGCAAGGAATTTTCCTGGGTTTAACAGGAGCAGAAACGTCGGAAATATGTCGCTGTCATCGACGGCCGACATGGC

Hap3 CCAATTCAACTGGCTCGATGCGTCAACCGTTGGCATCCTCCCACATGCAAGGAATTTTCCTGGGTTTAACAGGAGCAGAAACGTCGGAAATATGTCGCTGTCATCGACGGCCGACATGGC

Hap4 CCAATTCAACTGGCTCGATGCGTCAACCGTTGGCATCCTCCCACATGCAAGGAATTTTCCTGGGTTTAACAGGAGCAGAAACGTCGGAAATATGTCGCTGTCATCGACGGCCGACATGGC

Hap5 CCAATTCAACTGGCTCGATGCGTCAACCGTTGGCATCCTCCCACATGCAAGGAATTTTCCTGGGTTTAACAGGAGCAGAAACGTCGGAAATATGTCGCTGTCATCGACGGCCGACATGGC

Hap6 CCAATTCAACTGGCTCGATGCGTCAACCGTTGGCATCCTCCCACATGCAAGGAATTTTCCTGGGTTTAACAGGAGCAGAAACGTCGGATATATGTCGCTGTCATCGACGGCCGACATGGC

**************************************************************************************** *******************************

DQ869679_NAM-2_ TGGCGCGGGAACCTGCGCGGTGGACAACGGTGGAGGCAATGCGATGAACGTCATGCCTCCATTTATGAATCATCTCCCCGTGCAAGATGGGACCTACCATCAACAGCATGTCATCCTCGG

Hap1 TGGCGCGGGAACCTGCGCGGTGGACAACGGTGGAGGCAATGCGATGAACGTCATGCCTCCATTTATGAATCATCTCCCCGTGCAAGATGGGACCTACCATCAACAGCATGTCATCCTCGG

Hap2 TGGCGCGGGAACCTGCGCGGTGGACAACGGTGGAGGCAATGCGATGAACGTCATGCCTCCATTTATGAATCATCTCCCCGTGCAAGATGGGACCTACCATCAACAGCATGTCATCCTCGG

Hap3 TGGCGCGGGAACCTGCGCGGTGGACAACGGTGGAGGCAATGCGATGAACGTCATGCCTCCATTTATGAATCATCTCCCCGTGCAAGATGGGACCTACCATCAACAGCATGTCATCCTCGG

Hap4 TGGCGCGGGAACCTGCGCGGTGGACAACGGTGGAGGCAATGCGATGAACGTCATGCCTCCATTTATGAATCATCTCCCCGTGCAAGATGGGACCTACCATCAACAGCATGTCATCCTCGG

Hap5 TGGCGCGGGAACCTGCGCGGTGGACAACGGTGGAGGCAATGCGATGAACGTCATGCCTCCATTTATGAATCATCTCCCCGTGCAAGATGGGACCTACCATCAACAGCATGTCATCCTCGG

Hap6 TGGCGCGGGAACCTGCGCGGTGGACAACGGTGGAGGCAATGCGATGAACGTCATGCCTCCATTTATGAATCATCTCCCCGTGCAAGATGGGACCTACCATCAACAGCATGTCATCCTCGG

************************************************************************************************************************

DQ869679_NAM-2_ CGCCCCGCTCGCGCCAGAAGCCACCGGAGCCGCCGCCTCTGCCTTCCAGCATCCCGTTCAAATATCCGGCGTGAACTGGAATCCCTGA

Hap1 CGCCCCGCTCGCGCCAGAAGCCACCGGAGCCGCCGCCTCTGCCTTCCAGCATCCCGTTCAAATATCCGGCGTGAACTGGAATCCCTGA

Hap2 CGCCCCGCTCGCGCCAGAAGCCACCGGAGCCGCCGCCTCTGCCTTCCAGCATCCCGTTCAAATATCCGGCGTGAACTGGAATCCCTGA

Hap3 CGCCCCGCTCGCGCCAGAAGCCACCGGAGCCGCCGCCTCTGCCTTCCAGCATCCCGTTCAAATATCCGGCGTGAACTGGAATCCCTGA

Hap4 CGCCCCGCTCGCGCCAGAAGCCACCGGAGCCGCCGCCTCTGCCTTCCAGCATCCCGTTCAAATATCCGGCGTGAACTGGAATCCCTGA

Hap5 CGCCCCGCTCGCGCCAGAAGCCACCGGAGCCGCCGCCTCTGCCTTCCAGCATCCCGTTCAAATATCCGGCGTGAACTGGAATCCCTGA

Hap6 CGCCCCGCTCGCGCCAGAAGCCACCGGAGCCGCCGCCTCTGCCTTCCAGCATCCCGTTCAAATATCCGGCGTGAACTGGAATCCCTGA

****************************************************************************************
